# Enzymatic Synthesis of Biobased Polyesters and Polyamides

**DOI:** 10.3390/polym8070243

**Published:** 2016-06-25

**Authors:** Yi Jiang, Katja Loos

**Affiliations:** 1 Department of Polymer Chemistry, Zernike Institute for Advanced Materials, University of Groningen, Nijenborgh 4, 9747 AG Groningen, The Netherlands; y.jiang@rug.nl; 2 Dutch Polymer Institute (DPI), P.O. Box 902, 5600 AX Eindhoven, The Netherlands

**Keywords:** enzymatic polymerization, biobased polyesters, biobased polyamides, biobased monomer, lipase, renewable resources

## Abstract

Nowadays, “green” is a hot topic almost everywhere, from retailers to universities to industries; and achieving a green status has become a universal aim. However, polymers are commonly considered not to be “green”, being associated with massive energy consumption and severe pollution problems (for example, the “Plastic Soup”) as a public stereotype. To achieve green polymers, three elements should be entailed: (1) green raw materials, catalysts and solvents; (2) eco-friendly synthesis processes; and (3) sustainable polymers with a low carbon footprint, for example, (bio)degradable polymers or polymers which can be recycled or disposed with a gentle environmental impact. By utilizing biobased monomers in enzymatic polymerizations, many advantageous green aspects can be fulfilled. For example, biobased monomers and enzyme catalysts are renewable materials that are derived from biomass feedstocks; enzymatic polymerizations are clean and energy saving processes; and no toxic residuals contaminate the final products. Therefore, synthesis of renewable polymers via enzymatic polymerizations of biobased monomers provides an opportunity for achieving green polymers and a future sustainable polymer industry, which will eventually play an essential role for realizing and maintaining a biobased and sustainable society.

## 1. Polymers: From Petrol-Based to Biobased and Beyond

Polymers are one of the most important materials that are being exploited and developed by mankind, which play an essential and ubiquitous role in our modern life. They are large molecules or macromolecules that are composed of many small molecular fragments known as repeating units. They are in widespread use as plastics, rubbers, fibers, coatings, adhesives, foams and specialty polymers [1].

According to their origin, polymers can be classified as natural polymers or synthetic polymers. Natural polymers occur in nature via in vivo reactions, where biocatalysts, normally enzymes, are inevitably involved. Natural polymers can be found in all living organisms: plants, animals and human beings. Examples of natural polymers include lignocellulose, starch, protein, DNA, RNA and polyhydroxyalkanoates (PHAs), just to name a few. Normally, the structures of natural polymers are well-defined, with some exceptions like lignocellulose.

Synthetic polymers are commonly produced via polymerization of petrol-based chemicals having simple structures. Chemical catalysts, especially metal catalysts, are normally used in the preparation of synthetic polymers. Because of the booming of petrochemical industry and the concomitant availability of cheap petroleum oils, as well as the well establishment and advancement of polymerization techniques, numerous synthetic polymers have been developed, for example, phenol-formaldehyde resins, polyolefins, polyvinyl chloride, polystyrene, polyesters and polyamides, and so on. Synthetic polymers which include the large group known as plastics, became prominent since the early 20th century; and plastics are widely used as bottles, bags, boxes, textile fibers, films, and so on.

**Scheme 1:**
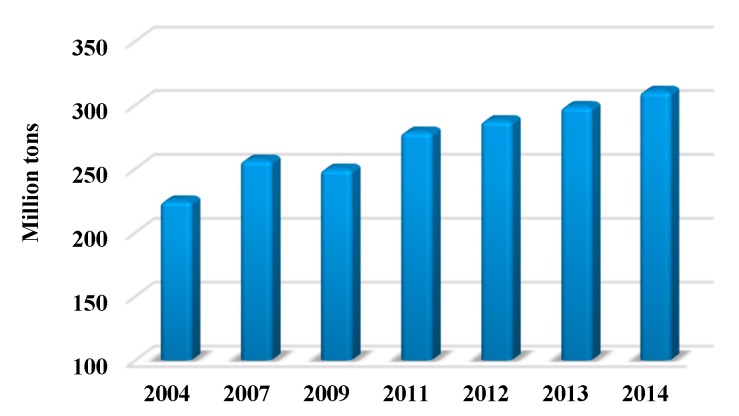
Global production of plastics from 2004 to 2014 [2].

Currently, there is a huge demand for polymers. The global production of plastics increased from 225 million tons in 2004 to 311 million tons in 2014 (Scheme 1) [2]; and the global polymer production is expected to reach 400 million tons in 2020 [3]. This huge polymer consumption leads to a massive demand for fossil resources for the polymer industry, which however brings some severe problems. On the one hand, fossil resources are depleting resources with limited storage; and their formation requires millions of years. There is a great concern that fossil resources will be exhausted within several hundred years. On the other hand, hazardous waste and emissions are generated along with the consumption of fossil resources, which induce severe environmental problems such as global warming and pollutions like smog and haze which are breaking out frequently, for instance in China nowadays. Driven by the growing environmental concerns, it is necessary and appealing to develop sustainable polymers for reducing the current dependence on fossil resources and decreasing the production of pollutants. As a matter of fact, laws have been approved by the European Union to reduce the usage of environmentally abusive materials, and to trigger more efforts to find eco-friendly materials based on renewable resources [4,5].

Biobased polymers are pointed out to be the most promising alternatives [5,6,7,8,9,10,11,12,13,14,15,16], which are defined as “sustainable materials for which at least a portion of the polymer consists of materials that are produced from renewable raw materials” [17]. Generally speaking, biobased polymers can be produced via three routes [8,11]: (1) pristine natural polymers, or chemical or physical modifications of natural polymers; (2) manufactured biobased polymers from a mixture of biobased molecules with similar functionalities that are converted from biomass feedstocks; and (3) synthesis of biobased polymers via polymerization of biobased monomers with tailored chemical structures.

Some natural polymers such as natural rubber, cotton, starch and PHAs, are useful materials; however, they are limited in variety, and their properties and applications are also limited as they are determined by their chemical structure. Considering the rich abundance of biomass feedstocks in nature, it is of great interest to produce biobased polymeric materials by chemical or physical modifications of natural polymers, or from biobased molecules that are converted from biomass feedstocks. Actually human beings already used the former approach long time ago during the 1800s. Many commercially important polymers are prepared via this approach, for example, vulcanized natural rubber, gun cotton (nitrocellulose), cellulose esters and cellulose ethers. However, chemical and physical modifications of natural polymers are often subject to the poor solubility and process difficulty of natural polymers, as well as, unwanted impurities within the network of natural polymers which are hard to remove. On the other hand, conversion of biomass feedstocks to end-products is a promising pathway for the production of high tonnage consumer polymeric products such as paper, paints, resins and foams [11]. For instance, oleochemicals can be converted from vegetable oils and fats, which are biobased building blocks for the production of thermoset resins and polyurethanes. However, the obtained biobased polymeric materials often possess diverse chemical structures; and it is nearly impossible to produce biobased polymers with identical structures as the petrol-based counterparts, due to the use of biomolecule mixtures. Besides, some unwanted structures or impurities might be inherited from the biomolecule mixtures, which might greatly influence the properties and applications of the final polymeric materials.

Utilization of biobased monomers with tailored structures in polymer synthesis is the most promising approach towards biobased polymers, which can result in not only sustainable alternatives to petrol-based counterparts with similar or identical structures, but also in novel green polymers that cannot be produced from petrol-based monomers [5,8,9,14,15,16]. However, this is also the most expensive approach of all three as aforementioned.

Benefiting from solar energy, numerous biobased monomers can be produced from yearly-based biomass feedstocks via biocatalytic or chemo-catalytic processes, which provide a great opportunity to access diverse biobased polymers [5,7,8,9,10,11,14,15,16,18,19,20,21,22,23,24,25,26,27]. Meanwhile, more and more biobased monomers are already or will become commercially available in the market due to the fast development of biotechnologies and their price will be competitive with that of the petrol-based chemicals [26,28,29,30,31,32,33,34].

Enzymatic polymerization is an emerging alternative approach for the production of polymeric materials, which can compete against conventional chemical synthesis and physical modification techniques [35,36,37,38,39,40,41,42,43,44]. Enzymatic polymerization also provides a great opportunity for accessing novel macromolecules that are not accessible via conventional approaches. Moreover, with mild synthetic conditions and renewable non-toxic enzyme catalysts, enzymatic polymerization is considered as an effective way to reduce the dependence of fossil resources and to address the high material consumption and pollution problems in the polymer industry.

At present, petrol-based monomers are still predominately used in enzymatic polymerizations. By combining biobased monomers and enzymatic polymerizations in polymer synthesis, not only the research field of enzymatic polymerization could be greatly accelerated but also the utilization of renewable resources will be promoted. This will provide an essential contribution for achieving sustainability for the polymer industry, which will eventually play an important role for realizing and maintaining a sustainable society.

## 2. Polyesters

**Scheme 2:**
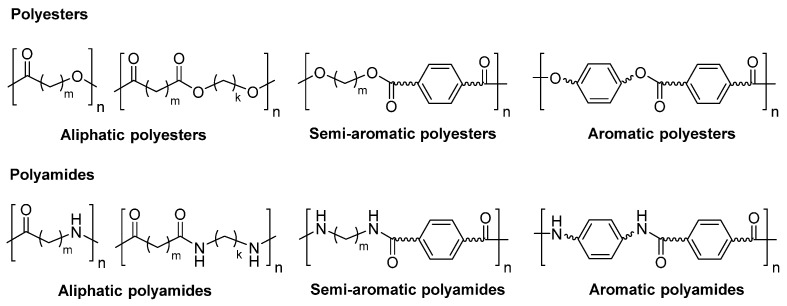
General chemical structures of aliphatic, semi-aromatic and aromatic polyesters and polyamides.

Polyesters are polymers in which the monomer units are linked together by ester groups. Examples of polyesters include some naturally occurring polyesters like cutin, shellac, and poly (hydroxybutyrate) (PHB), and many synthetic polyesters such as poly(butylene succinate) (PBS), poly(lactic acid) (PLA), poly(ethylene terephthalate) (PET), polybutylene terephthalate (PBT) and poly(4-hydroxybenzoate-*co*-6-hydroxynaphthalene-2-carboxylic acid) (Vectran^®^, Kuraray, Chiyoda-ku, Tokyo, Japan). According to the chemical composition of the main chain, polyesters can be classified as aliphatic, semi-aromatic and aromatic polyesters (Scheme 2).

Most known aliphatic polyesters could be produced as biobased polymers [45,46], as the majority of their starting monomers can be produced from biomass feedstocks. Aliphatic polyesters are also (bio)degradable materials which can be recycled, disposed, composted or incinerated with a low environmental impact [46,47]. Aliphatic polyesters are widely used as thermoplastics and thermoset resins, with many commodity and specialty applications. Among them, PLA is the most well-known aliphatic polyester, which can be used as fibers, food packaging materials and durable goods, with a global demand of around 360 kilo tons in 2013 [48]. PBS is another important commodity polyester which can be applied as packaging films and disposable cutlery, with a global market of around 10–15 kilo tons per year [49]. In addition, aliphatic polyesters have found potential applications in biomedical and pharmaceutical fields such as in sutures, bone screws, tissue engineering scaffolds, and drug delivery systems, due to their biodegradability, biocompatibility and probable bioresorbability [46,50,51,52].

Compared to aliphatic polyesters, semi-aromatic polyesters generally possess better thermal and mechanical properties, which can be used as commodity plastics and thermal engineering plastics. Examples of semi-aromatic polyesters are poly(trimethylene terephthalate) (PTT), PET, PBT, and poly(ethylene naphthalate) (PEN). Among them, PET is the most commonly used semi-aromatic polyester. It is the fourth-most-produced plastic [53], with a global supply of more than 19.8 million tons in 2012 [54]. PET has been widely used as beverage bottles, food containers, fibers and fabrics, packing films, photographic and recording tapes, engineering resins, and so on. It should be noted that PET is commonly referred by its common name, polyester, in textile and fiber applications; whereas the acronym “PET” or “PET resin” is used when applied as bottles, containers and packaging materials.

Aromatic polyesters are high performance thermoplastics, with high thermal stability and chemical resistance, and excellent mechanical properties. Aromatic polyesters have found many applications in the mechanical, chemical, electronic, aviation and automobile industries [55]. However, aromatic polyesters generally possess a poor solubility even in aggressive solvents and are difficult to process, caused by their extremely rigid structures [56]. Examples of aromatic polyesters are poly(4-hydroxybenzoate-*co*-6-hydroxynaphthalene-2-carboxylate) (Vectra^®^, Celanese, Irving, TX, USA; Vectran^®^, Kuraray, Chiyoda-ku, Tokyo, Japan), poly(4-hydroxybenzoate-*co*-4,4′-biphenylene terephthalate) (Xydar^®^, Solvay, Brussels, Belgium; Ekonol^®^, Saint-Gobain, Courbevoie, France) and poly(6-hydroxynaphthalene-2-carboxylate-*co*-4-hydroxybenzoate-*co*-4,4′-biphenylene terephthalate).

Besides, aromatic polyesters and some semi-aromatic copolymers such as poly(2-chlorohydroquinone terephalate-*co*-l,4-cyclohexylenedimethylene terephthalate) and poly(*p*-hydroxybenzoate-*co*-ethylene terephthalate) are liquid crystalline materials in which both liquid crystalline and polymer properties are combined. These liquid crystalline polyesters are generally characterized by a rod-like molecular structure, rigidness of the long axis, and strong dipoles [55]. Aromatic polyesters are good candidates for thermotropic main-chain polymers due to the highly rich aromatic (mesogenic) fragments, and the low inter-chain forces because of the relatively low energy of association of the ester groups.

Generally speaking, polyesters can be produced via two methods: (1) step-growth polycondensation of diols and diacid/diesters, or hydroxyacids/hydroxyesters; and (2) ring-opening polymerization of cyclic monomers (lactones, cyclic diesters and cyclic ketene acetals) and cyclic oligomers. Both of these two methods have some merits and also suffer from some drawbacks. On the one hand, the building blocks for step-growth polycondensation are generally easily obtained at a relatively cheap price. However, elevated reaction temperatures (150–280 °C), long reaction times, high vacuum condition, heavy metal catalysts and a precise stoichiometric balance between monomers are normally required for polycondensation. In addition, side-reactions and volatilization of monomers may occur at elevated temperatures or under high vacuum [50,57]. On the other hand, removal of by-products is not required by ring-opening polymerization and, therefore, high molecular weight products can be obtained under relatively mild conditions in a matter of minutes. Besides, side reactions can be greatly suppressed during ring-opening polymerization. However, extra synthesis steps and heavy metal catalysts are often required for the preparation of the starting materials, cyclic monomers and cyclic oligomers.

Moreover, polyesters can be also synthesized by other methods such as polyaddition of diepoxides to diacids [58], and acyclic diene metathesis (ADMET) polymerization of diene monomers containing ester bonds in the main chain [59].

At present, some biobased polyesters are already commercially available, including fully biobased PLA, PHAs, and poly(ethylene furanoate) (PEF), partially biobased PBS, PET, PTT and poly(butylene adipate-*co*-terephthalate) (PBAT), and so on (Table 1) [34,49,60,61,62,63,64,65,66,67,68]. However, polymers including polyesters, polyamides and other types, are still mainly derived from petroleum oils. The production capacity of biobased polymers represented only a 2% share of the total polymer production in 2013 and will increase to 4% by 2020 [3].

## 3. Polyamides

Polyamides are polymers in which the monomeric units are linked together by amide bonds. Examples of polyamides include naturally occurring polyamides like proteins, and synthetic polyamides such as polycaprolactam (nylon 6 or PA 6), poly(hexamethylene adipamide) (nylon 6,6 or PA 6,6), poly(hexamethylene terephathamide) (PA 6,T), and poly(*p*-phenylene terephathamide) (PPTA, Kevlar^®^, DuPont, Wilmington, DE, USA). Similar to polyesters, polyamides can be classified to three types: aliphatic, semi-aromatic and aromatic polyamides, depending on the chemical composition of the main chain (Scheme 2).

Aliphatic polyamides, commercially known as nylons or nylon fibers, are highly valued semi-crystalline thermoplastics that are widely used as synthetic fibers, construction materials, food packing materials, engineering resins, and so on [69]. Currently, a variety of aliphatic polyamides are commercially manufactured, including nylon 6 (PA 6), nylon 10 (PA 10), nylon 11 (PA11) and nylon 12 (PA 12), and nylon 4,6 (PA 4,6), nylon 6,6 (PA 6,6), nylon 6,10 (PA 6,10) and nylon 6,12 (PA 6,12). Among them, nylon 6 is the largest produced aliphatic polyamide by far, with a global production of 4.2 million tons in 2010; and nylon 6,6 ranked the second largest aliphatic polyamide in the market, with a global production of 2.1 million tons. Actually, nylon 6,6 is the first example of aliphatic polyamides, which was firstly produced in the laboratory by Carothers and Hill at DuPont in 1930. After that, this polyamide was prepared by DuPont as nylon 6,6 fiber on 28 February 1935, and then produced at full-scale in July 1935. Regarding nylon 6, it was firstly developed by Schlack at IG Farbenindustrie in 1938, for the purpose of reproducing the properties of nylon 6,6 without violating the patents [70,71]. At present, 66% of nylon 6 production is used as fibers, 30% is applied as engineering thermoplastics, and the rest 10% is consumed as films. For nylon 6,6, 55% of the current production is used as fibers, and the remainder is applied as engineering thermoplastics. Other nylons like nylon 4,10, nylon 6,12, nylon 10,10, nylon 11 and nylon 12 are commonly used as high performance materials [72].

Semi-aromatic polyamides consist of both aliphatic and aromatic fragments in the polymer main chain. Especially, polyphthalamides (PPAs), a type of semi-aromatic polyamides, are defined by ASTM D5336 as “polyamides in which at least 55 mol % of the carboxylic acid portion of the repeating unit in the polymer chain is comprised by a combination of terephthalic acid (TPA) and isophthalic acid (IPA)” [73]. Compared to aliphatic polyamides, semi-aromatic polyamides are much stiffer, rendering the polyamides with higher mechanical strength and better thermal resistance. In addition, semi-aromatic polyamides possess many other merits such as high heat chemical/abrasion/corrosion resistance, good dimensional stability, superior processing characteristics and direct bonding to many elastomers. Semi-aromatic polyamides can be used as thermal engineering materials and high performance materials, which have found various applications in many areas, for example, in marine, automotive industry, oil industry, electronics, machinery, domestic appliances, medical devices, personal care, and so on. Examples of semi-aromatic polyamides are PA 6,T, poly(nonamethylene terephthalamide) (PA 9,T), and poly(decamethylene terephthalamide) (PA 10,T). They are commercially produced by many companies such as DuPont (Zytel^®^HTN, PA 6,T), Solvay (Amodel^®^, PA 6,T), EMS-GRIVORY (Grilamid^®^HT, PA 6,T), Mitsui (ARLEN^®^, PA 6,T/6,6), Kuraray (Genesta^®^, PA 9,T), and Evonik (VESTAMID^®^HTplus, PA 6,T/X or PA 10,T/X) [72,74].

Aromatic polyamides are normally referred to wholly aromatic polyamides, or aramids in which at least 85% of the amide linkages are directly attached to two aromatic groups [73,75,76]. Due to the amide linkages and the rigid aromatic structures, the stiff rod-like aromatic polyamide chains interact with each other by strong and highly directional hydrogen bonds and π-π stackings. Therefore, aromatic polyamides possess outstanding thermal and mechanical resistance, and excellent chemically inert property, but a poor solubility and processability. Aromatic polyamides are high performance materials that are used as advantageous replacement for metals or ceramics, cut-resistant, flame resistant and high-tensile strength synthetic fibers and coatings, bullet-proof body armor, protective clothing, electrical insulation materials, sealing materials, composites, and so on. Examples of aromatic polyamides are PPPTA and poly(*m*-phenylene isophthalamide) (PMPI). These two aramids are the most well-known commercially available aromatic polyamides, with the trademark of Kevlar^®^ (DuPont) and Nomex^®^ (DuPont), respectively. Besides, some aromatic polyamides display liquid crystalline properties. For example, the solid-state PPPT (Kevlar^®^, DuPont, Wilmington, DE, USA) is an example of main chain lyotropic liquid crystal polymers [55].

Similar to polyesters, polyamides can be generally synthesized via two methods: (1) step-growth polycondensation of diacids/diesters with diamines, or ω-amino acids/esters; and (2) ring-opening polymerization of lactams. For example, nylon 6,6 is produced by polycondensation of adipic acid and 1,6-hexanediamine, while nylon 6 is typically produced by ring-opening polymerization of ε-caprolactam.

Regarding the equipment and the reaction conditions followed, the polymerization steps in polyester and polyamide synthesis are similar [57]. However, with respect to the formation of high molecular weight products, the polymerization of polyamides differs from that of polyesters to some extent. Firstly, the chemical equilibrium is favored for the amide formation but is less favored for the ester formation. Secondly, when dicarboxylic acids are used as starting materials, salts are formed in polyamide synthesis, but there is no salt formation in polyester synthesis. In this case, stoichiometric equivalence can be much more easily achieved in polyamide synthesis. Thirdly, the amide interchange reactions (transamidations) are much slower than the ester interchange reactions (transesterifications).

Currently, some biobased polyamides are already commercially available, including fully biobased nylon 4,10, nylon 10,10 and nylon 11, and partially biobased nylon 6,10, nylon 10,12 and PA 10,T, and so on (Table 2).

## 4. Biobased Monomers for Polyester and Polyamide Synthesis

Generally speaking, lactones, diacids and their ester and anhydride derivatives, diols, polyols, and hydroxyacids and their esters are good building blocks for polyester synthesis, while lactams, ω-amino acids and their esters, diacids and their derivatives, and diamines are suitable monomers for polyamide synthesis. Herein, some predominate biobased monomers for polyester and polyamide synthesis are outlined.

### 4.1. Biobased Lactones and Lactams

Lactones and lactams are abundant moieties in naturally occurring compounds with diversified structures and varied ring sizes. Examples of naturally occurred lactones and lactams are tetronic acid, 5,6-dihydropyran-2-one, coumarin, α-alkylidene-γ-lactones and lactams, α-alkylidene-δ-lactones and lactams, β-lactam, and so on. They are widely applied in the fine and functional perfumery and in the pharmaceutical industry. However, few studies referred to the synthesis of polyesters and polyamides from naturally occurring lactones and lactams, probably due to their complicated structures, limited availability, and high price [77].

3-Hydroxybutyrolactone (3-HBL) is a biobased platform molecules listed in “DOE TOP 10” [18]. It is a chiral compound that can be used for the synthesis of pharmaceuticals, polymers and organic solvents. However, the chemical synthesis of 3-HBL is quite difficult, with multiple steps [5,18,78]. Currently, (*S*)-3-HBL is commercially produced from l-malic acid via a continuous chemical synthesis process under high pressure in a fixed-bed reactor using a ruthenium-based catalyst [79,80] This process involves hazardous conditions, expensive catalysts, as well as multiple purification steps [78]. Recently, Prather et al. [78,81] developed a biosynthesis pathway for 3-HBL in recombinant *E. coli* (*Escherichia coli*) using glycolic acid or glucose as the starting material. However, the large-scale biological production of 3-HBL is still challenging, which requires further studies.

Some other lactones, such as propiolactone, γ-butyrolactone, angelilactone, γ-valerolactone, and furan-2(5H)-one, can be derived from renewable resources [18,82].

**Scheme 3:**
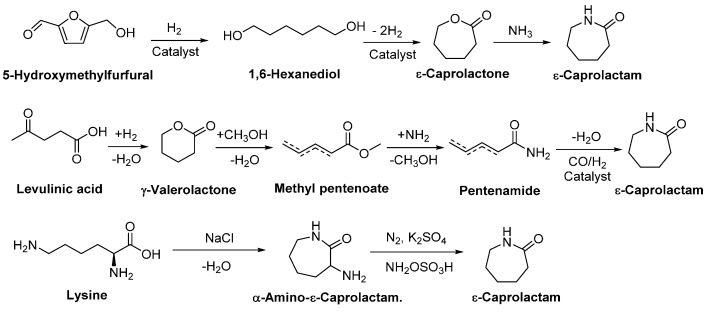
Synthesis of ε-caprolactam from biobased chemicals.

In addition, lactams can be converted from biomass feedstocks. Among them, ε-caprolactam is an important raw material for the synthesis of nylon 6. At present, ε-caprolactam is produced via a six-step chemical process using benzene and ammonia as starting materials. Recently Heeres et al. [83] reported the conversion of biobased 5-(hydroxymethyl)furfural (HMF) to ε-caprolactam via four steps (Scheme 3), two steps less than the traditional approach. In addition, Bouwman et al. [84] reported the production of ε-caprolactam from biobased levulinic acid via a four-step process. Moreover, synthesis of ε-caprolactam from sugar-derived lysine is developed [85,86]. It is also possible to produce ε-caprolactam via fermentation of sugars and the relevant industrial process is currently under development [31].

### 4.2. Biobased Aliphatic Diacids

Succinic acid is a naturally occurring dicarboxylic acid, which is predominantly produced commercially through petrochemical routes by catalytic hydrogenation of maleic acid or anhydride, with a global production of 30–50 kilo tons per year [87,88]. Succinic acid can be also produced by fermentation of carbohydrates or glycerol using engineered bacteria or yeast. The current bio-route for succinic acid is based on proprietary *E. coli* or yeast strains [88]. To lower the cost, other microorganisms and yeast have been developed, like *Coryne*-type bacteria, which shows a significantly higher productivity compared to *E. coli*. [33] Currently, four companies have built up commercial facilities for the production of biobased succinic acid: Reverdia, Succinity, Bioamber and Myriant [29].

Itaconic acid is an attractive unsaturated monomer that has already been produced industrially by sugar fermentation using *Aspergillus terreus* early in the 1960s [89,90]. The current production of itaconic acid is around 80 kilo tons per year, mainly in USA, China, Japan and France [91]. To reduce the cost and increase the sustainability, current studies mainly focus on strain improvement of microorganisms by mutagenesis, development of more cost-effective process methodologies, and the use of alternative cheap substrates such as cellulolytic biomass [91].

Adipic acid is one the most important commodity chemicals, which is mainly used for the production of nylon 6,6 [33,92]. The current global market for adipic acid is around 4 million tons per year [31]. At present, over 90% of adipic acid is manufactured industrially by oxidation of cyclohexanol or KA-oil (a mixture of cyclohexanol and cyclohexanone) using concentrated nitric acid [92,93,94,95]. In recent years, two prospective biosynthetic pathways to biobased adipic acid have been developed and are under commercialization evaluation at the moment [31,33]: (1) chemo-catalytic conversion of biologically derived precursors such as *cis*,*cis*-muconic acid or d-glucaric acid; and (2) direct biological conversion of vegetable oils and sugars using yeast.

In addition, suberic acid, sebacic acid and dodecanedioic acid are also (potentially) biobased monomers which can be converted from plant oils [31,96,97,98].

### 4.3. Biobased Aliphatic Diols and Polyols

1,3-Propanediol (1,3-PDO) is a commodity chemical used for the production of various polymers, with an annual global demand of around 1 million tons [99]. At present, there are two chemical processes for the industrial production of 1,3-PDO, starting from petrol-based acrylaldehyde or ethylene oxide [100,101]. Nowadays biobased 1,3-PDO is commercially synthesized via fermentation of d-glucose based on corn using a genetically engineered *E. coli*. [100] In addition, it is promising to produce 1,3-PDO from biomass-derived glycerol using a bacterial fermentation process [99,100,101,102,103].

1,4-Butanediol (1,4-BDO) is widely used as a building block for polymer synthesis, with an annual global market of over 2.5 million tons [104]. The industrial production of 1,4-BDO dominantly depends on petrol-based chemicals such as maleic anhydride, acetylene, butane, propylene and butadiene. Since late 2007, Genomatica (USA) started to develop a biological process for the synthesis of biobased 1,4-BDO from sugars using a genetically-modified strain of *E*. *coli* bacteria [99,104,105,106]. This process has already been commercialized [31]. Alternatively, biobased 1,4-BDO can be produced by reduction of sugar-derived succinic acid and this process is under commercialization preparation stage [31].

**Scheme 4:**
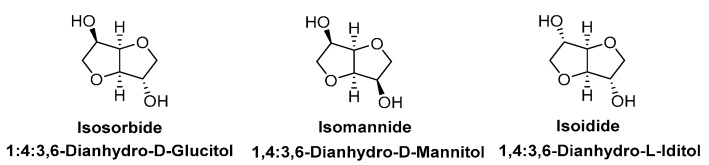
Chemical structures of 1,4:3,6-dianhydrohexitols (DAHs).

1,4:3,6-Dianhydrohexitols (DAHs) are sugar-derived aliphatic diols with rigid and chiral structures [107]. It is of great interest to synthesize DAH-based polymers with high glass transition temperatures (*T*_g_) and/or with special optical properties [108]. According to the chirality, DAHs have three possible stereoisomers: isosorbide, isomanide and isoidide (Scheme 4). Due to the different positions of the hydroxyl groups, the reactivity of these isomers are different, showing the following sequence: isomannide < isosorbide < isoidide [107,108]. Nowadays, only isosorbide is produced at an industrial scale using sugars as the starting materials [26,107]; and Roquette (France) is a leading producer. However, the purity and high price of the commercial isosorbide are two major concerns when used for polyester synthesis.

Other aliphatic diols such as 2,3-butanediol, 1,6-hexanediol, 1,8-octanediol and 1,10-decanediol, are (potentially) biobased monomers [5,109,110].

Moreover, glycerol and d-sorbitol are abundant and inexpensive biobased aliphatic polyols. Glycerol is obtained as a byproduct in the production of biodiesel from vegetable oils and fats [5,111], while d-sorbitol is produced industrially on large scale by reduction of glucose derived from biomass feedstocks [33].

Furthermore, sugars like glucose and sucrose, and sugar alcohols such as erythritol, xylitol and sorbitol, are polyols with multi hydroxyl groups. They are naturally occurring compounds which can be produced via fermentation of various sources of biomass feedstocks [112].

### 4.4. Biobased Aliphatic Diamines

1,4-Butanediamine (1,4-BDA, putrescine) is naturally produced by decomposition of amino acids in living and dead organisms, which is used for the production of engineering plastics and high performance materials such as nylon 4,6, nylon 4,10 and PA 4,T. It is produced industrially via chemical synthesis approaches starting from petrol-based 1,4-dichloro-2-butene or 1,4-dihalobutane, or succinodinitrile [113,114]. It is also possible to synthesize biobased 1,4-BDA via chemical conversion of biomass-derived succinic acid [18], or via fermentation of sugars using engineered *E. coli* strains [113].

1,5-Pentanediamine (1,5-PDA, cadaverine) is a naturally occurring compound which is produced by hydrolysis of protein during the tissue putrefaction of animals, the same as 1,4-BDA. 1,5-PDA can be used for the production of nylon 5,6 and nylon 5,10. The industrial production of 1,5-PDA is similar to that of 1,4-BDA, using petrol-based 1,5-dichloropentane, glutarodinitrile, or glutaraldehyde as the starting material [114]. Moreover, the biosynthesis of 1,5-PDA is well established, by decarboxylation of lysine using several microorganisms [115,116]. It is also promising to produce biobased 1,5-PDA via fermentation of sugars by metabolic engineering. Currently, biobased 1,5-PDA has been produced in industrial scale by Cathay Industrial Biotech (Shanghai, China) [29]. In addition, Ajinomoto (Tokyo, Japan) is working on the industrial production of biobased 1,5-PDA by decarbonating of lysine via an enzymatic process.

1,6-Hexanediamine (1,6-HDA) is a raw material for synthesis of nylon 6,6, nylon 6,10 and PA 6,T, which is currently produced industrially from petrol-based butadiene or propylene. Recent developments show that biobased 1,6-HDA can be produced by chemical-catalytic conversion of adipic acid [117] or 1,6-hexanediol [110] derived from carbohydrates, or by a fermentation route [31]. The commercial production of biobased 1,6-HDA is already in preparation stage [31].

1,8-Octanediamine (1,8-ODA) can be potentially derived from biomass. It can be produced by amination of suberic acid which can be converted from plant oils [118].

1,10-Decanediamine (1,10-DDA) can be chemically converted from sebacic acid derived from castor oils. They are interesting biobased monomers for the synthesis of fully biobased nylon 10,10 which have already been commercially available in the market [31].

### 4.5. Biobased Aromatic Monomers

**Scheme 5:**
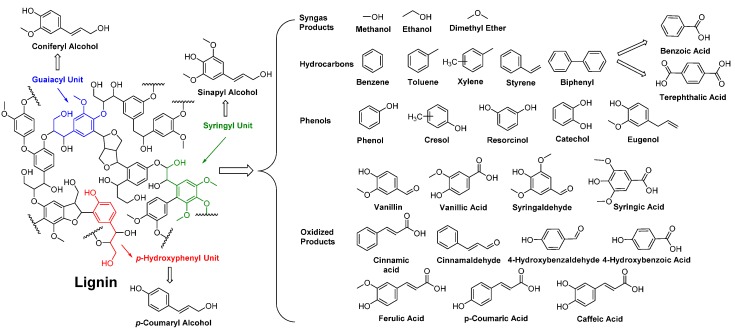
Promising biobased chemicals derived from lignin.

Lignin is the largest non-carbohydrate components of lignocellulosic biomass which is composed by oxidative coupling of three phenylpropane components: *p*-coumaryl alcohol, coniferyl alcohol, and sinapyl alcohol [119]. Due to the unique structures and chemical properties, lignin provides a broad opportunity for the production of a wide variety of biobased chemicals, especially biobased aromatic chemicals that so far cannot be accessible via chemical or biological modifications of other biomass feedstocks (Scheme 5).

However, it remains a big challenge to develop an efficient approach for the recovery of aromatic chemicals with tailored structures from lignin [120]. Currently, only vanillin can be produced via a commercial process by oxidation of lignosulfonates, a byproduct from the sulfite pulping industry [121,122,123]. Recently, new chemical and biotechnological approaches for the production of vanillin are studied [23,120,124,125,126]. Starting from vanillin, many biobased aromatic monomers for polyester synthesis can be produced, for example, vanillic acid, divanillyl diol, dimethyl divanillate, and so on [127,128,129,130,131].

**Scheme 6:**
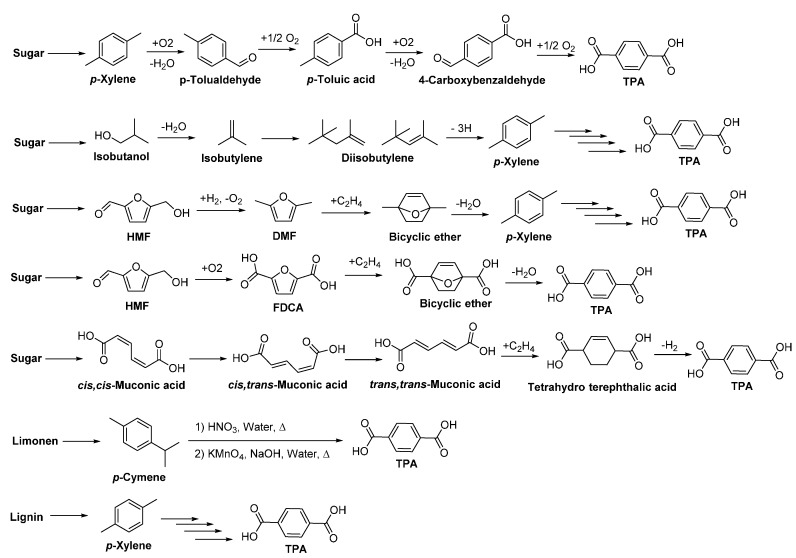
Proposed routes to biobased terephthalic acid (TPA).

Terephthalic acid (TPA) is industrially produced by oxidation of *p*-xylene. It is used mainly as a precursor for the production of aromatic polyesters and polyamides such as PET, PBT and PPAs. The current global market size of TPA is around 30 million tons per year, and is expected to increase to 60 million tons in 2020 [31]. Nowadays, several technologies to produce biobased TPA and its precursors from renewable resources have been proposed (Scheme 6) [49,132,133,134,135,136,137,138,139]; and some companies and research institutes are active in the development of biobased TPA [31,49,138] and full biobased PET. Nevertheless, no commercial biobased TPA and fully biobased PET are current available in the market.

**Scheme 7:**
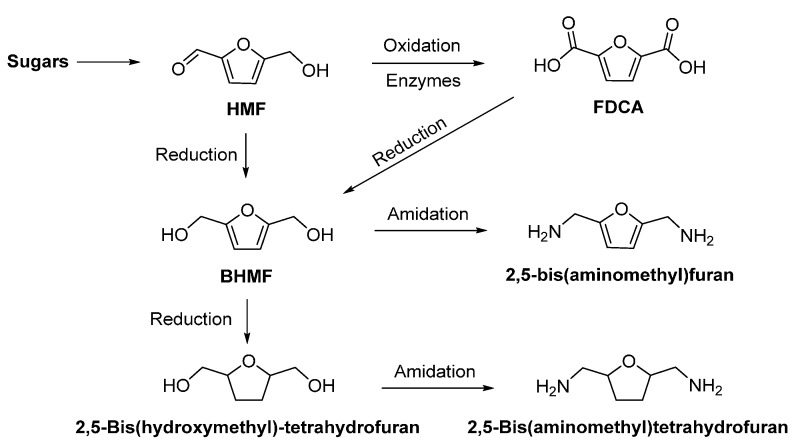
Promising biobased furan monomers for polyester or polyamide synthesis.

2,5-Furandicarboxylic acid (FDCA) is an interesting biobased rigid monomer, which is considered as the most promising substitute to petrol-based TPA and IPA [5,14,140]. Currently, FDCA is readily produced from biomass feedstocks, for example, by oxidation of HMF derived from various sources of carbohydrates [18,24]. It is also possible to produce FDCA via a biocatalytic approach starting from HMF (Scheme 7) [141]. At present, FDCA is industrially produced by Avantium (The Netherlands) using an enabling chemical synthesis technology [14,31]; and the price is expected to be cheaper than the biobased and petrol-based TPA [31,142].

Other interesting biobased furan monomers for polyester or polyamide synthesis include 2,5-bis(hydroxymethyl)furan (BHMF), 2,5-bis(aminomethyl)furan, 2,5-bis(hydroxymethyl) -tetrahydrofuran and 2,5-bis(aminomethyl)tetrahydrofuran (Scheme 7) [5,18,24,143].

### 4.6. Other Biobased Monomers

Lactic acid, one of the most well-known organic acids occurring naturally, can be found in many carbohydrates, for example, in naturally and fermented food products, plant, human beings and animals [144]. In most living organisms, lactic acid is also identified as a principal metabolic intermediate. Lactic acid can be manufactured chemically or biologically in industry [144]. In the chemical synthesis approach, lactic acid is prepared via hydrolysis of lactonitrile, a by-product of acrylonitrile production, by concentrated hydrochloric or sulfuric acid. This process is simple, but results in a racemic mixture of d- and l-lactic acid; and the production of lactic acid depends on the acrylonitrile industry in this case [145]. On the other hand, lactic acid can be produced via fermentation of sugars by bacteria. This microbial fermentation process involves the utilization of biomass feedstocks, low reaction temperature, low energy consumption and can resulted in enantio-pure lactic acid by selecting an appropriate microbial strain [145,146,147]. Currently, the global demand of lactic acid is 350 kilo ton per year, with a sustainable growth in the next decade; and more than 90% of lactic acid is commercially produced via fermentation of glucose [59]. Alternatively, production of lactic acid from biobased glycerol and its derivatives is feasibly; however, this route cannot compete with the fermentation process because of the high cost.

3-Hydroxypropionic acid (3-HPA) is a valuable biobased platform building blocks listed in “DOE TOP 10” and revised “DOE TOP 10” [18,22]. It can be produced via chemical approaches staring from 1,3-PDO, 3-hydroxypropionaldehyde or acrylic acid, which are not cost-effective [5]. In recent years, promising biosynthetic pathways have been developed to produce biobased 3-HPA, for example, via fermentation of sugars using genetically modified microorganisms [148,149,150]. Currently, the commercial production of biobased 1,3-HPA is under preparation stage by several companies including Perstorp, Opxbio-Dow chemical, BASF-Cargilland-Novozymes, and Metabolix [29].

Many long chain fatty acids and their derivatives can be produced from renewable resources such as plant oils and fats [97,151,152,153], and they are good building blocks for polyester and polyamide synthesis. Examples of long chain fatty acids include oleic acid, ricinoleic acid, erucic acid, vernolic acid, and so on [9,154].

Moreover, there are many other biobased building blocks for polyester or polyamide synthesis, such as ethylene glycol [31], polycarboxylic acids (citric acid, tartaric acid) [8], 11-amino-undecanoic acid [31], and so on.

## 5. Lipases

**Scheme 8:**
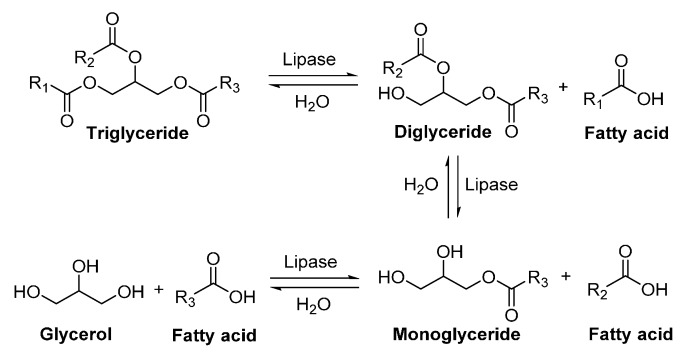
Lipase-catalyzed hydrolysis of triglyceride.

Lipases (triacylglycerol lipases, triacylglycerol acyl hydrolases, E.C. 3.1.1.3) are enzymes which catalyze the hydrolysis of water-insoluble triglycerides with long-chain fatty acids to di-glycerides, mono-glycerides and glycerol with release of free fatty acids in aqueous solution (Scheme 8). In organic synthesis, lipases can be used to catalyze other reactions in non-aqueous media, for example, esterification, transesterification, interesterification, amidation, transamidation, aminolysis, aldol condensation and Michael addition [155,156,157,158,159].

Generally, lipases possess high catalytic reactivity in nonpolar organic solvents with log *P* (logarithm of partition coefficient) of more than 1.9 [160,161,162]. Examples of suitable organic solvents for lipases are benzene (2), toluene (2.5), diphenyl ether (4.05), hydrocarbons like cyclohexane (3.2) and *n*-hexane (3.5), and so on [163]. Lipases also function in some green solvents such as ionic liquids and supercritical CO_2_ [164,165,166,167,168].

**Scheme 9:**
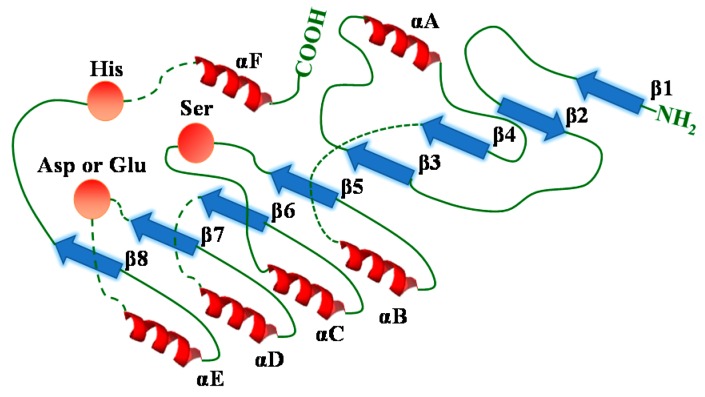
Secondary structure diagram of the α/β hydrolase fold and the location of catalytic triad amino acid residues in lipases. Ser: serine residue; Asp or Glu: aspartic or glutamic acid residue; His: histidine residue; helixes indicate α-helixes; arrows indicate β-sheets [177].

Despite their different sources and diverse structures, all lipases possess a very similar α/β hydrolase fold (Scheme 9). The α/β hydrolase fold consists of a β-sheet core of five to eight parallel strands (only the second β strand shows an antiparallel orientation to the others) connected on both sides by α-helices, forming a α/β/α sandwich-like shape [169,170,171,172]. Lipases and other enzymes including esterases, proteases, dehalogenases, epoxide hydrolases and peroxidases which exhibit similar structural features, belong to the α/β hydrolase family [169,173].

It is generally acknowledged that the specificity, selectivity and catalytic reactivity of an enzyme depend on its active site, the region that undergoes the binding of substrate molecules and the occurrence of enzymatic reactions. The active site of an enzyme consists of amino acid residuals that form temporary bonds with the substrate (binding site) and other amino acid residues that catalyze the corresponding reaction of that substrate (catalytic site). As for lipases, the active site is situated inside a pocket, which is located above the central β-sheet of the protein [174]. Although the active sites of lipases have different shapes, sizes, depths of the pockets, and physicochemical characteristics of their amino acids [175], the binding sites display highly homologous amino acid sequences [171]; and the active site of lipases consists of a highly conserved catalytic triad: a nucleophilic residue (serine), a histidine base and a catalytic acidic residue (aspartic or glutamic acid, usually aspartic acid) (Scheme 9). In addition, many lipases exhibit a lid, a surface loop that is a lipophilic α-helical domain in the polypeptide chain and covers the active sites [171,176]. The lid controls the access of substrate molecules to the catalytic center of lipase. In the presence of a lipid-water interface, the lid opens the active center and thus the active site becomes accessible. In this case, a large hydrophobic surface of the enzyme is revealed, which activates the enzyme. However, without the lipid-water interface, the lid is in a closed confirmation. As a consequence, the active center is not accessible and the enzyme is inactive.

**Scheme 10:**
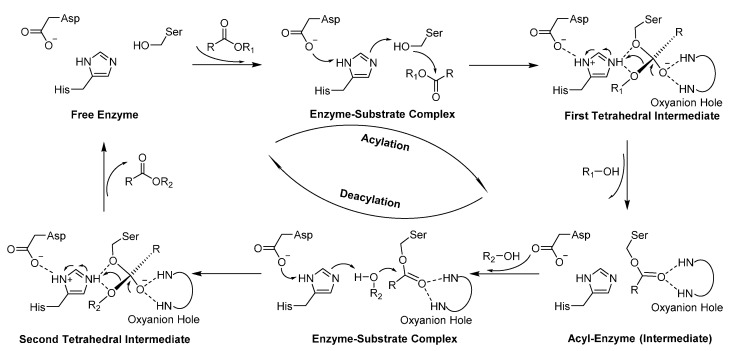
General catalytic mechanism of lipases [174].

The general catalytic mechanism of lipases is illustrated in Scheme 10, which involves an acylation step followed by a deacylation step [171,174]. At the acylation step, the hydroxyl group of the catalytic serine is activated by transferring a proton among the aspartate, histidine, and serine residues of the catalytic triad, rendering an increase of the nucleophilicity of the hydroxyl residue of the serine. After that, the hydroxyl residue of the serine attacks the carbonyl group of the substrate (carboxylic ester or carboxylic acid), forming the first tetrahedral intermediate with a negative charge on the oxygen of the carbonyl group. The oxyanion hole is formed by hydrogen bonding between the amide groups of the amino acid residuals of the enzyme and the carbonyl group oxygen of the substrate. By the formation of at least two hydrogen bonds in the oxyanion hole, the charge distribution is stabilized and the state energy of the tetrahedral intermediate is reduced. Then the alcohol component (R_1_–OH) is released from the bond with the intermediate, while the “acidic component” of the substrate remains covalently bound to the serine residue in the acyl-enzyme intermediate. When the enzyme is attacked by a nucleophile (R_2_–OH), the deacylation step occurs. The product (a new carboxylic ester or carboxylic acid) is then released, while the enzyme is regenerated. This nucleophile (R_2_–OH) can be water (hydrolysis) or an alcohol (alcoholysis).

To increase the stability towards organic solvents and to facilitate the recycling and reusing, lipases are normally used in their immobilized forms [178,179,180,181,182,183,184,185]. The immobilized lipases may show improved catalytic activity, specificity or selectivity. Similar to other enzymes, lipases can be generally immobilized via three strategies [178]: (1) chemical or physical adsorptions onto an inert matrix; (2) entrapment within an inert matrix; and (3) immobilized as water-insoluble particles: cross-linked enzyme aggregates (CLEAs), cross-linked enzyme crystals (CLECs), and protein-coated microcrystals (PCMC).

**Scheme 11:**
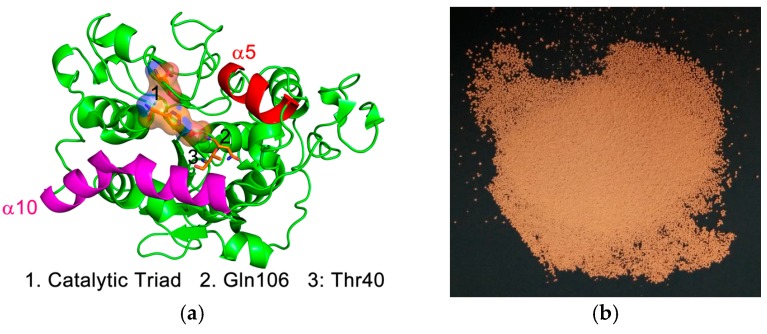
(**a**) The crystal structure of *Candida antarctica* lipase b (PDB number: 1TCA, from http://www.rcsb.org/); and (**b**) a photo of Novozym^®^ 435 beads.

Due to the broad substrate specificity, high selectivity, and high thermal stability and catalytic reactivity, *Candida antarctica* lipase b (CALB), which was reclassified as *Pseudozyma antarctica* lipase b (PALB) more recently [186], is the most popular biocatalyst which is extensively used in biocatalytic synthesis of small molecules and polymers. CALB is a globular protein that is composed of 317 amino acids (Scheme 11), having a molecular weight of 33 kDa. Similar to other lipases, CALB possesses a Ser-His-Asp catalytic triad (Ser105, Asp187 and His224) in its active site and two oxyanion holes (Thr40 and Gln106) [187], and the catalytic mechanism of CALB is the same as other lipases.

However, the presence of the lid structure and the interfacial activation of CALB are still under debate. Some literature suggested that the two α-helixes (α5 and α10) surrounding the active center of CALB, the most mobile part of the structure, could work as the lid [188,189,190,191], and CALB is an interfacial activated enzyme. A recent study indicated the hydrophobicity of the interface and the overall size of the substrate determine the interfacial activation of CALB [190], Others suggested that CALB has no lid covering the entrance of the active site [187] and displays no interfacial activation [192]. In addition, CALB has a very limited available space in the pocket of active site compared to other lipases and this explains its high selectivity [193].

CALB shows improved thermal stability and more stable performance in its immobilized form. At present, several immobilized CALB formulations are commercially available, including Novozym^®^ 435 (N435, Novozymes A/S, Copenhagen, Denmark), Chirazyme^®^ l-2 (Roche Molecular Biochemicals, Mannheim, Germany), LCAHNHE and LCAME (SPRIN S.p.A, Milano, Italy), and CalB immo Plus™ (c-LEcta, Leipzig, Germany, and Purolite, Bala Cynwyd, PA, USA) [194]. The immobilized CALB formulations are currently frequently used in industry, for example, for the synthesis of pharmaceutical chiral intermediates, and for the production of other value-added fine chemical compounds.

N435 is the primary immobilized CALB that is used both in the industrial area and academia research. N435 functions as a hydrophobic biocatalyst, which consists of 10 wt % of CALB physically absorbed within 90 wt % of Lewatit VP OC 1600 bead which is a macroporous DVB-crosslinked methacrylate polymer resin [162,194,195]. The bead size of N435 ranges from 0.315 to 1.0 mm (>80%), the effective size is around 0.32–0.45 mm, and the average pore diameter is 15 nm. N435 can work at mild conditions and especially, can tolerate some extreme conditions such as elevated temperatures (up to 150 °C) [196,197,198].

## 6. Enzyme-Catalyzed Synthesis of Polyesters

Enzymatic polymerization is defined as “in vitro (in the test tubes) chemical synthesis of polymers via a non-biosynthetic (non-metabolic) approach using an isolated enzyme as the catalyst” [36,199].

Due to the unique properties of enzymes, enzymatic polymerization inherits many merits such as high specificity and selectivity towards monomer substrates, clean-process, energy saving, gentle environmental footprint, nontoxic natural catalysts, and recyclable catalysts (after immobilization). With these, enzymatic polymerization provides an opportunity to achieve “green polymer chemistry”.

At present, 4 enzyme classes, oxidoreductases, transferases, hydrolases and ligases, are identified to induce or catalyze polymerizations (Table 3) [200]. Many polymers are successfully synthesized via enzymatic polymerizations, for example, vinyl polymers [38,201], polysaccharides [202,203,204,205], polyesters [42,44] and polyamides [206,207,208]. Among them, polyesters are the most extensively studied polymers in enzymatic polymerization; and lipases are the most efficient biocatalysts for enzymatic polymerization of polyesters [42].

**Scheme 12:**
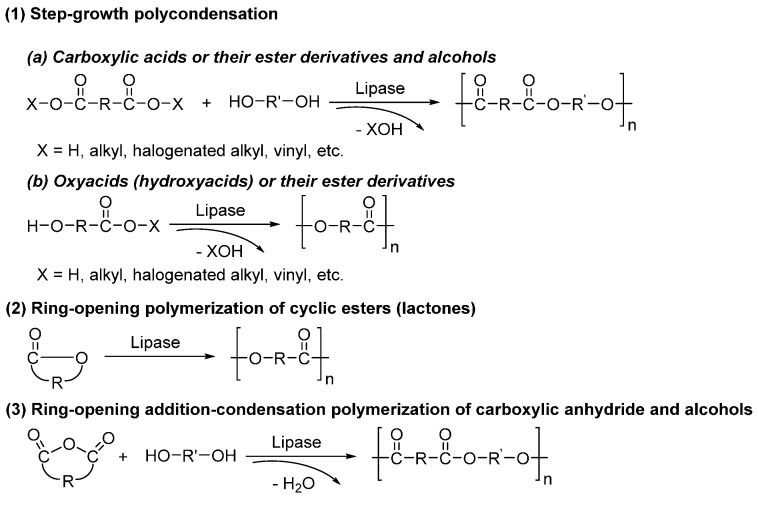
Main reaction modes of lipase-catalyzed synthesis of polyesters.

Generally speaking, three polymerization modes can be proceeded for the lipase-catalyzed polyester synthesis (Scheme 12): (1) step-growth polycondensation; (2) ring-opening polymerization; and (3) a combination of ring-opening polymerization and polycondensation (ring-opening addition-condensation polymerization). Among them, polycondensation and ring-opening polymerization are the most common methods used for biocatalytic polyester synthesis.

**Scheme 13:**
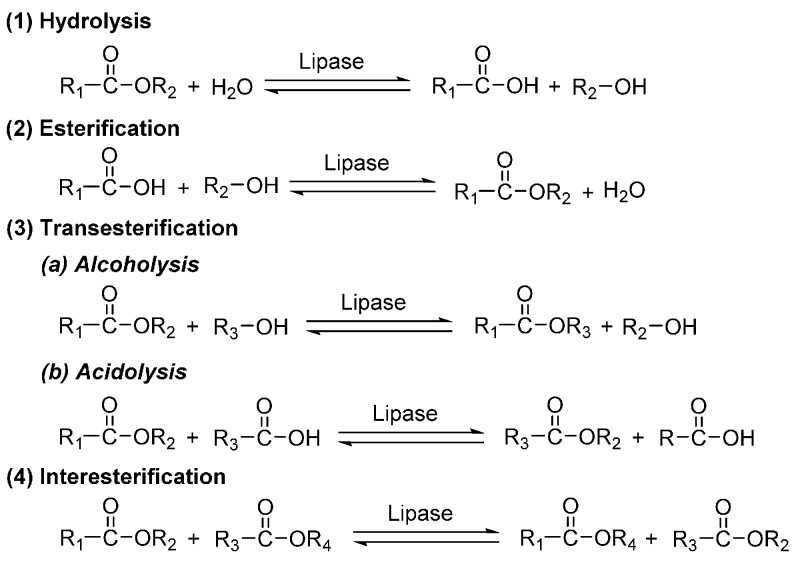
Basic modes of elemental lipase-catalyzed reactions in biocatalytic polyester synthesis.

Four modes of elemental reactions may occur during the lipase-catalyzed polyester synthesis, inducing hydrolysis, esterification, transesterification (alcoholysis and acidolysis), and interesterification (Scheme 13). These reactions are all reversible. Therefore, to facilitate the ester formation, it is crucial to remove the remaining water and byproducts like alcohols from the reaction mixture, for example, by adding absorbing and drying agents like molecular sieves, applying reduced pressure, using azeotropic distillation conditions, and so on.

**Scheme 14:**
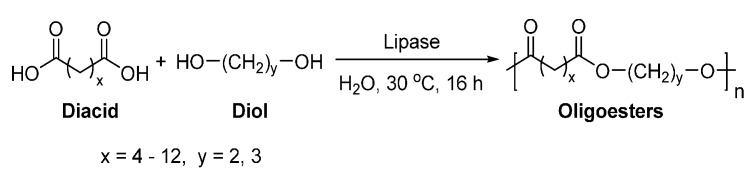
Lipase-catalyzed polycondensation of aliphatic diacids and diols.

The first lipase-catalyzed polymerization was reported by Okumara et al. in 1984 [209]. They investigated the enzymatic polymerization of aliphatic diacids and diols by a lipase from *Aspergillus niger* NRRL 337 (Scheme 14). However, only oligoesters with $\bar{M_{n}}$’s of around 1000 g/mol were obtained.

**Scheme 15:**
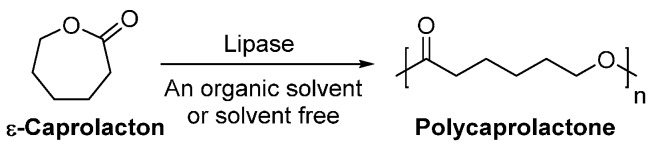
Lipase-catalyzed ring-opening polymerization of ε-caprolactone.

The lipase-catalyzed ring-opening polymerization was firstly reported in 1993 by two independent groups [210,211]. Gutman et al. [210] investigated the lipase-catalyzed ring-opening polymerization of ε-caprolactone (ε-CL); and polycaprolactone (PCL) with a $\bar{M_{n}}$ of up to 4400 g/mol was successfully produced in *n*-hexane (Scheme 15). At the same time, the enzymatic ring-opening polymerization of lactones was performed in bulk by Kobayashi et al. [211], using different lipases as catalysts. The enzymatic polymerization gave PCL and polyvalerolactone with $\bar{M_{n}}$’s of up to 7700, and 1900 g/mol, respectively.

In the late 1990s, the use of N435 in the enzymatic ring-opening polymerization of lactones was introduced by Gross et al. [212] Since then, N435 became the working horse in biocatalytic polyester synthesis.

After these pioneer works, various combinations of monomer substrates such as diacids/diesters and diols, hydroxyacids/esters, and cyclic monomers like lactones, cyclic diesters and cyclic ketene acetals, are studied for the lipase-catalyzed polymerization. The recent progress in this field is comprehensively summarized in some review articles [35,36,40,41,42,44,213,214].

**Scheme 16:**
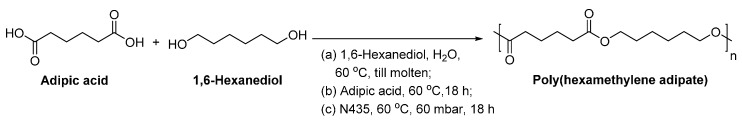
N435-catalyzed synthesis of poly(hexamethylene adipate) in large scale, using a two-stage method.

It should be pointed out that the large scale production of aliphatic polyesters via lipase-catalyzed polymerization is feasible. As reported by Binns et al. [215], adipic acid and 1,6-HDO were polymerized by N435 at a multi-kilogram scale, using a two-stage method (Scheme 16). The enzymatic polymerization yielded poly(hexamethylene adipate) with a $\bar{M_{w}}$ of 16,400 g/mol. They also claimed that the enzymatic production can be scaled up to the pilot plant level (2.0 tons) without undue problems. Besides, poly(hexamethylene adipate) produced from the enzymatic polymerization possesses a lower acid number, higher degree of crystallinity and super crystalline growth rate compared to the conventional counterparts.

Moreover, macrolides catalyzed by lipases showed higher polymerizability compared to smaller ring-sized lactones [216]. This is probably because macrolides possess higher rates in the formation of enzyme-activated monomers (acyl-enzyme intermediates). However, reverse tendency was observed from anionic and metal (Zn) catalyzed-ring opening polymerization.

Although a great number of aliphatic polyesters are readily synthesized with high molecular weights via lipase-catalyzed polymerization, only limited amount of semi-aromatic and aromatic polyesters are enzymatically produced [217,218,219,220,221,222,223,224,225,226,227]. This could be mainly due to the high melting temperature (*T*_m_) of semi-aromatic and aromatic polyesters and their low solubility in the reaction media, as well as, the lack of reactivity of aromatic monomers in enzymatic polyesterification [210,228]. However, by using cyclic aromatic oligomers in the lipase-catalyzed polymerization, high molecular weight poly(alkylene terephthalate)s, poly (alkylene isophthalate)s and poly(benzenedimethanol adipate)s were obtained, with $\bar{M_{w}}$’s of up to 107,000 g/mol [229].

## 7. Enzyme-Catalyzed Synthesis of Polyamides

Lipases, proteases and other enzymes are capable of catalyzing the formation of amide bonds and therefore, they are suitable enzymes for the in vitro polyamide synthesis [206]. In the following discussion of this section, we focus on the lipase-catalyzed polymerization of synthetic polyamides.

Similar to the biocatalytic polyester synthesis, the lipase-catalyzed polyamide synthesis can proceed via three basic modes: (1) step-growth polycondensation of diacid/diesters and diamines or ω-amino carboxylic acids/esters; (2) ring-opening polymerization of lactams; and (3) a hybrid of step-growth polycondensation and ring-opening polymerization.

**Scheme 17:**
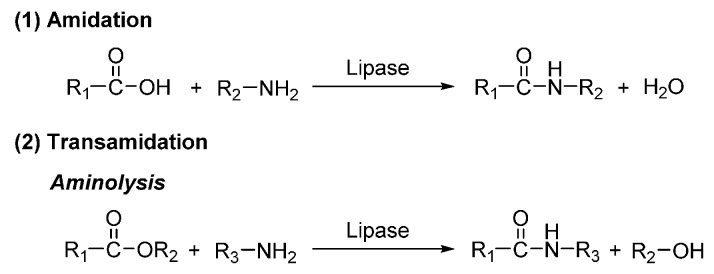
Basic modes of elemental lipase-catalyzed reactions in biocatalytic polyamide synthesis.

Two basic modes of elemental reactions are commonly used in the biocatalytic polyamide synthesis: directly amidation and transamidation (aminolysis) (Scheme 17).

The lipase-catalyzed polymerization of polyamides has not been well studied [208]. This could be attributed mainly to two reasons: (1) the high *T*_m_ of polyamides, and (2) the poor solubility of polyamides in common organic solvents. On the one hand, polyamides like nylons and TPA-based polyamides are semi-crystalline polymers which normally possess a high *T*_m_ above 100 °C. At such elevated temperatures, the catalytic reactivity of lipases is significantly decreased due to the occurrence of protein denaturation and deactivation. On the other hand, many polyamides can be only dissolved in some aggressive solvents such as formic acid, concentrated H_2_SO_4_, and trifluoroacetic acid, in which lipases cannot act.

Nevertheless, some oligoamides and polyamides are successfully produced via the lipase-catalyzed polymerization [206,207,208]. Some typical examples are given below.

**Scheme 18:**
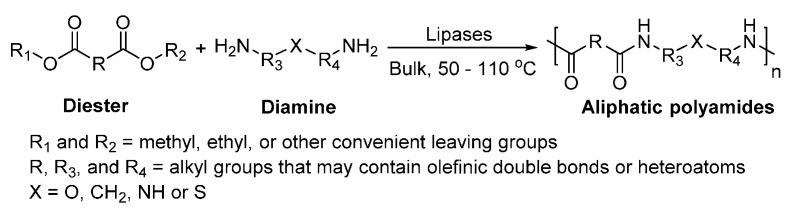
Lipase-catalyzed synthesis of aliphatic polyamides.

Cheng et al. [230,231] investigated the lipase-catalyzed polymerization of diamines and diesters in bulk (Scheme 18), which resulted in aliphatic polyamides with $\bar{M_{w}}$’s of around 3000–15,000 g/mol. This is the first report showing that high molecular weight polyamides can be produced from lipase-catalyzed polymerization.

The CALB-catalyzed ring-opening polymerization of ε-caprolactam was reported by Kong et al. [232]. They claimed that the enzymatic ring-opening polymerization gave nylon 6 with a high $\bar{M_{w}}$ of 212,000 g/mol.

**Scheme 19:**
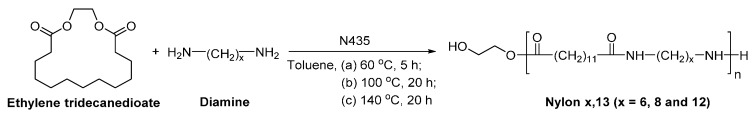
N435-catalyzed ring-opening addition-condensation of ethylene tridecanedioate with various diamines.

Aliphatic polyamides such as nylon 6,13, nylon 8,13 and nylon 12,13 were synthesized via the N435-catalyzed ring-opening addition-condensation (Scheme 19) [197]. The $\bar{M_{n}}$’s of the resulting nylons were around 5600–8300 g/mol.

**Scheme 20:**
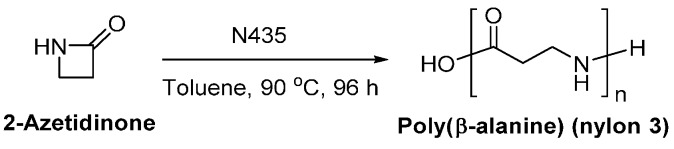
Enzymatic ring-opening polymerization of 2-azetidinone.

In our group, enzymatic polymerization of polyamides is one of the focused research area. For example, the enzymatic polymerization of 2-azetidinone was first studied in our laboratory (Scheme 20) [233]. A different mechanism for the enzymatic ring-opening polymerization of β-propiolactam was revealed and a catalytic cycle for the oligomerization of β-lactam that rationalizes the activation of the monomers was proposed [234]. Moreover, aliphatic oligoamides [233,235,236], semi-aromatic oligoamides [237], and poly(ester amide)s [238] are successfully prepared via lipase-catalyzed polymerization in our laboratory.

## 8. Lipase-Catalyzed Synthesis of Sustainable Polyesters and Polyamides from Biobased Monomers

At present, most research on enzymatic polymerization still focused on the use of “traditional” monomers derived from fossil resources. Due to the growing awareness of energy safety and environmental pollution, and increased interest for the development of novel polymeric materials, utilization of biobased monomers in enzymatic polymerization becomes an appealing topic both in the academic and industrial fields. Currently, many (potentially) biobased polyesters and polyamides are readily synthesized via enzymatic polymerization. In this section, the recent developments in the field of the lipase-catalyzed synthesis of biobased polyesters and synthetic polyamides are discussed in details.

### 8.1. Biobased Saturated Aliphatic Polyesters

#### 8.1.1. Poly(lactic acid)

**Scheme 21:**
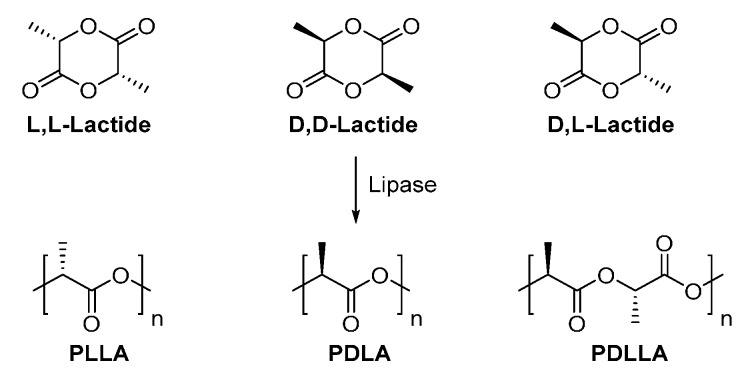
l,l-, d,d- and d,l-lactide, and lipase-catalyzed ring-opening polymerization of lactides.

PLA is commonly produced by ring-opening polymerization of lactides or by direct polycondensation of lactic acid, using chemical catalysts. It is also possible to synthesize PLA via lipase-catalyzed ring-opening polymerization (Scheme 21).

However, the direct enzymatic ring-opening polymerization of lactides generally resulted in PLA with low molecular weights or low reaction yields, indicating that the enzymatic polymerization efficiency was quite low. It was also found that the enzymatic polymerization of d,l-lactide resulted in higher molecular weight products compared to d,d- and l,l-lactide [239,240,241]. Nevertheless, after careful adjusting the reaction conditions, high molecular weight poly(d,d-lactide) (PDLA) can be synthesized from the N435-catalyzed ring-opening polymerization, with a $\bar{M_{n}}$, dispersity and conversion of 12,000 g/mol, 1.1 and 60%, respectively [242]. In addition, poly(l,l-lactide) (PLLA) can be produced from the N435-catalyzed ring-opening polymerization in supercritical CO_2_ [243]. Although the resulting PLLA possessed a high $\bar{M_{w}}$ (12,900 g/mol) and a good dispersity (around 1.2), the reaction yield was quite low, less than 12%.

**Scheme 22:**
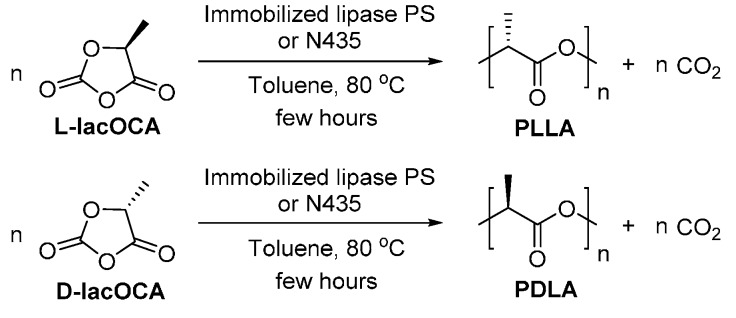
Immobilized lipase Amano PS-C II from *Pseudomonas sp.* (lipase PS) or N435-catalyzed ring-opening polymerization of an *O*-carboxylic anhydride derived from lactic acid (l- or d-lacOCA) [244].

A new biocatalytic approach was developed for the efficient synthesis of high molecular weight PLLA and PDLA, starting from an *O*-carboxylic anhydride derived from lactic acid (l- or d-lacOCA) (Scheme 22) [244]. The $\bar{M_{n}}$, dispersity and reaction yield of the resulting PLLA and PDLA were up to 38,400 g/mol, ≤1.4, and around 90%, respectively. In addition, the tested lipases showed slight preference to l-lacOCA over d-lacOCA. Moreover, the molecular weights of the obtained PLLA can be controllable by altering the concentration of N435 in the reaction media.

By employing alcohol initiators containing different hydroxyl numbers (2, 4, 6, 8, and 22), various linear and branched PLAs were synthesized via the *Pseudomonas fluoresaens* lipase-catalyzed ring-opening polymerization of l,l-, d,d-, and d,l-lactide in bulk [245]. The enzymatic polymerization yielded PLAs with $\bar{M_{n}}$’s and dispersities of around 1500–36,700 g/mol, and 1.0–1.5, respectively.

**Scheme 23:**
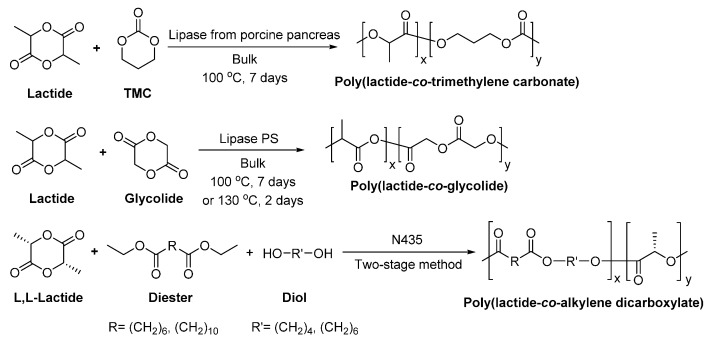
Enzymatic *co*-polymerization with lactides [246,247,248].

Moreover, many PLA *co*-polyesters were successfully synthesized via lipase-catalyzed *co*-polymerization, including poly(lactide-*co*-trimethylene carbonate) [246], poly(lactide-*co*-glycolide) [247], and poly(lactide-*co*-alkylene dicarboxylate) [248] (Scheme 23). The corresponding $\bar{M_{w}}$‘s were around 12,000–21,000, 2200–20,600, and 10,000–38,000 g/mol, respectively. Besides, the obtained *co-*polyesters can be optically active, due to the retention of the chiral configuration of lactate units after the enzymatic polymerization [248].

#### 8.1.2. Poly(butylene succinate)

PBS is normally synthesized via polycondensation of succinic acid or succinic anhydride with 1,4-BDO at elevated temperatures, using a chemical catalyst [249]. It is also promising to synthesize biobased PBS via enzymatic polymerization.

**Scheme 24:**
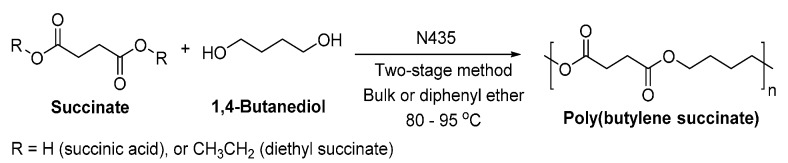
Enzymatic polycondensation of succinic acid or diethyl succinate and 1,4-butanediol, using a two-stage method [250].

The lipase-catalyzed polycondensation of PBS was studied by Gross et al. [250], using a two-stage method which is similar to those used for the industrial production but at much lower temperatures (Scheme 24). The solvent-free enzymatic polycondensation with succinic acid gave oligomers. However, by replacing succinic acid with diethyl succinate, the temperature varied two-stage method in diphenyl ether resulted in PBS with a $\bar{M_{w}}$ of 38,000 g/mol and a dispersity of 1.39.

**Scheme 25:**
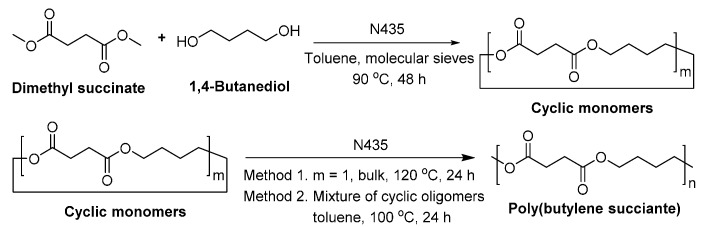
N435-catalyzed synthesis of poly(butylene succinate) by cyclization with subsequent ring-opening polymerization of the cyclic oligomers [251].

**Scheme 26:**
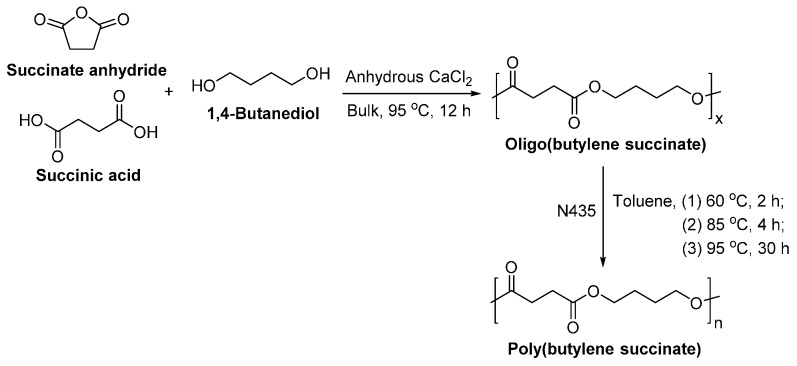
Synthesis of poly(butylene succinate) via N435-catalyzed *co*-polymerization of succinic acid and 1,4-butanediol with succinate anhydride [252].

To synthesize PBS with higher molecular weights, another two enzymatic strategies were developed: (1) using cyclic oligomers [251]; and (2) *co*-polymerization of succinic acid and 1,4-BDO with succinate anhydride [252]. By using cyclic butylene succinate oligomers in the N435-catalyzed polymerization, PBS with a $\bar{M_{w}}$ of up to 130,000 g/mol and a dispersity of 1.6 was obtained. However, under similar reaction conditions, the direct enzymatic polycondensation gave PBS with a lower $\bar{M_{w}}$ (45,000 g/mol) and a broader dispersity (3.7) (Scheme 25). On the other hand, the enzymatic *co*-polymerization of succinic acid and 1,4-BDO with succinate anhydride resulted in PBS with a $\bar{M_{w}}$ of 73,000 g/mol and a dispersity of 1.7 (Scheme 26). However, although high molecular weight PBS can be enzymatically produced via these two approaches, an extra synthesis step is required.

#### 8.1.3. Other Biobased Aliphatic Polyesters

Many other (potential) biobased aliphatic polyesters are synthesized via lipase-catalyzed polycondensation. Some examples are discussed as follows.

The lipase-catalyzed solvent-free polycondensation of aliphatic diacids (C2–C12) and aliphatic diols (C2–C12) was performed by Kobayashi et al. [253] The enzymatic polymerization yielded various aliphatic polyesters with $\bar{M_{n}}$’s and dispersities of around 1300–14,000 g/mol, and 1.1–2.3, respectively.

**Scheme 27:**
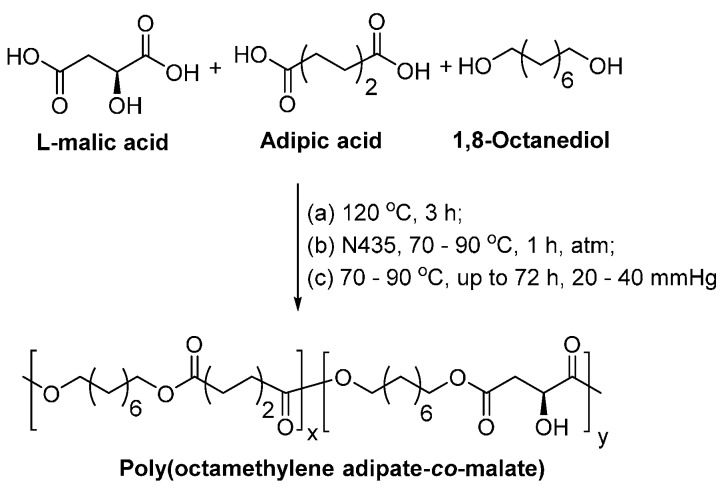
Enzymatic synthesis of poly(octamethylene adipate-*co*-malate) using N435 as the biocatalyst [254].

Biodegradable *co*-polyesters containing malic acid units were synthesized via the N435-catalyzed polycondensation of adipic acid and 1,8-octanediol with l-malic acid, a natural occurring monomer (Scheme 27) [254]. The solvent-free enzymatic polycondensation gave poly(octamethylene adipate-*co*-malate), with $\bar{M_{w}}$’s, dispersities and reaction yields of 4700–9500 g/mol, 1.50–1.92, and 88–96%, respectively.

The lipase-catalyzed polymerization of aliphatic diacid ethyl esters (C2, C4 and C8) and diols (C4, C6 and C8) were performed in bulk or by using β-cyclodextrin as the support architecture. Various saturated aliphatic polyesters were produced, with $\bar{M_{w}}$’s ranging from 5300 to 44,600 g/mol [255,256].

**Scheme 28:**
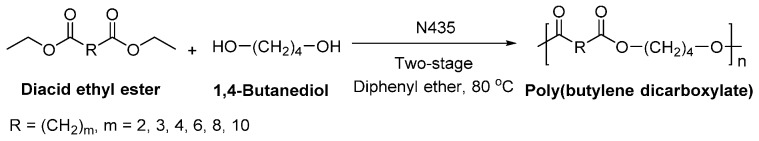
Enzymatic synthesis of poly(butylene dicarboxylate)s via N435-catalyzed polycondensation of 1,4-butanediol and diacid ethyl esters differing in chain length [257].

Recently, we succeeded in preparing a series of (potentially) biobased poly(butylene dicarboxylate)s via the N435-catalyzed polycondensation of 1,4-butanediol and diacid ethyl esters differing in chain length (C2, C3, C4, C6, C8 and C10) (Scheme 28) [257]. High molecular weight poly(butylene dicarboxylate)s were obtained, with $\bar{M_{w}}$’s of up to 94,000 g/mol. We found that increasing the chain length of diacid ethyl ester from C2 to C4 resulted in poly(butylene dicarboxylate)s of significant higher molecular weights; however, upon further increasing the chain length from C4 to C10, poly(butylene dicarboxylate)s with lower molecular weights were obtained. Meanwhile, the enzymatic polymerization with diethyl succinate (C2) gave the lowest molecular weight products. This suggested that CALB possesses a higher selectivity towards diacid ethyl esters with a >C2 chain length; and CALB prefers diethyl adipate (C4) over the other tested counterparts.

Moreover, biobased telechelic polyesters were produced via N435-catalyzed polycondensation of azelaic acid and 1,6-HDO with different functional end-cappers in supercritical CO_2_ [258]. The $\bar{M_{n}}$, dispersity and reaction yield of the resulting telechelic poly(hexamethylene azelate)s were around 1500–2400 g/mol, 1.73–2.18, and 78%–88%, respectively. The obtained telechelic polyesters can be further modified by cross-linking or by chain extension reactions.

### 8.2. Biobased Unsaturated Aliphatic Polyesters

Currently, the synthesis of biobased unsaturated polyesters, especially itaconate-based unsaturated polyesters, has not been well studied. This is because the sensitive C=C bond can be deteriorated easily under conventional polymerization conditions such as elevated temperatures and metal catalysts. However, this problem can be easily overcome by using enzyme catalysts in the polymerization, due to the mild synthetic conditions and the high catalytic specificity of the enzyme catalysts.

**Scheme 29:**
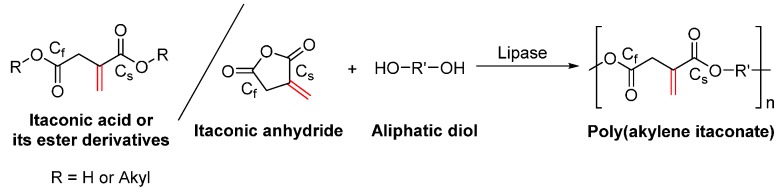
Enzymatic polymerization of itaconate derivatives and aliphatic diols.

However, the lipase-catalyzed direct polycondensation of itaconate and aliphatic diols with short chain length generally resulted in oligomers. As reported by Gardossi et al., the solvent-free polyesterification of dimethyl itaconate and 1,4-BDO catalyzed by CALB gave a mixture of oligomers from dimer to pentamer [259,260]. Similarly, the N435-catalyzed polymerization of itaconic anhydride with aliphatic diols (C4–C10) gave oligomers with $\bar{M_{n}}$’s of around 150–390 g/mol, although itaconic anhydride was completely consumed [261]. This is because the enzymatic polycondensation is hampered by the low reactivity of itaconate due to the lower electrophilicity of the acyl carbon (C_s_, Scheme 29) adjacent to the vinyl group [259]. However, the low reactivity of itaconate in enzymatic polymerization could be overcome by optimizing the reaction conditions: (1) improving the mass transfer and the enzyme distribution in the reaction mixture; (2) increasing the enzyme loading; (3) lowering the diol concentration; and (4) choosing more appropriate diols [259].

Indeed, by using glycols with longer chain lengths or with a rigid structure, itaconate-based homo-polyesters with relatively higher molecular weights were obtained from the lipase-catalyzed polycondensation. As reported by Yousaf et al. [262], the N435-catalyzed polymerization of itaconic acid with 1,4-cyclohexanedimethanol/poly(ethylene glycol) gave homo-polymers with a $\bar{M_{w}}$ of 2600 and 8600 g/mol, respectively. On the contrary, the tin(II) 2-ethylhexanoate-catalyzed polycondensation with itaconic acid gelled within hours.

In addition, itaconate-based *co*-polyesters with high molecular weights can be prepared via lipase-catalyzed *co*-polymerization, as discussed below.

The N435-catalyzed *co*-polymerization of itaconic acid, adipic acid and 3-methyl-1,5-pentanediol resulted in a *co*-polymer with a $\bar{M_{w}}$ of 19,000 g/mol [262].

**Scheme 30:**
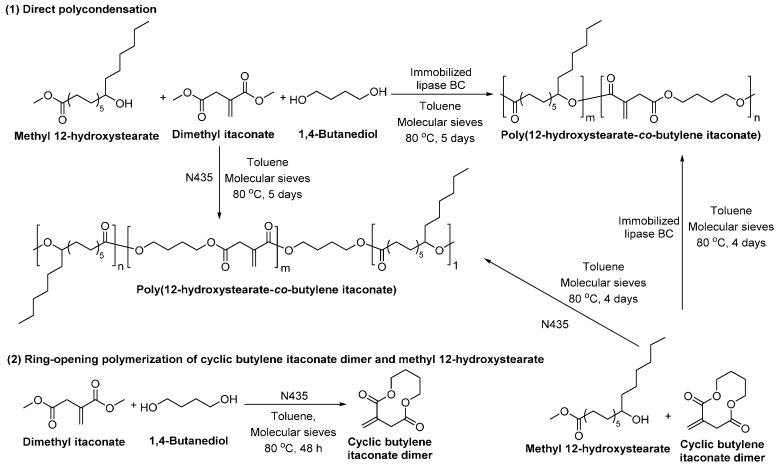
Lipase-catalyzed synthesis of poly(12-hydroxystearate-*co*-butylene itaconate), using N435 or immobilized *Burkholderia cepacia* lipase (lipase BC) as the catalyst.

Poly(12-hydroxystearate-*co*-butylene itaconate) with a $\bar{M_{w}}$ of 30,000 g/mol was obtained from the lipase-catalyzed directly polycondensation of methyl 12-hydroxystearate, dimethyl itaconate and 1,4-BDO (Scheme 30) [263]. Moreover, the lipase-catalyzed ring-opening addition-condensation polymerization of methyl 12-hydroxystearate and cyclic butylene itaconate dimer resulted in poly(12-hydroxystearate-*co*-butylene itaconate) with a significantly higher $\bar{M_{w}}$ of 160,000 g/mol. Furthermore, the NMR study indicated that the enzymatic polymerization catalyzed by different lipases yielded poly(12-hydroxystearate-*co*-butylene itaconate) with different microstructures. As shown in Scheme 30, no ester bond was formed between the hydroxyl group of 12-hydroxystearate and the carboxyl group of itaconate when the polymerization was catalyzed by N435. However, by using immobilized *Burkholderia cepacia* lipase (lipase BC), an ester bond was formed between the 12-hydroxystearate and itaconate unit.

**Scheme 31:**
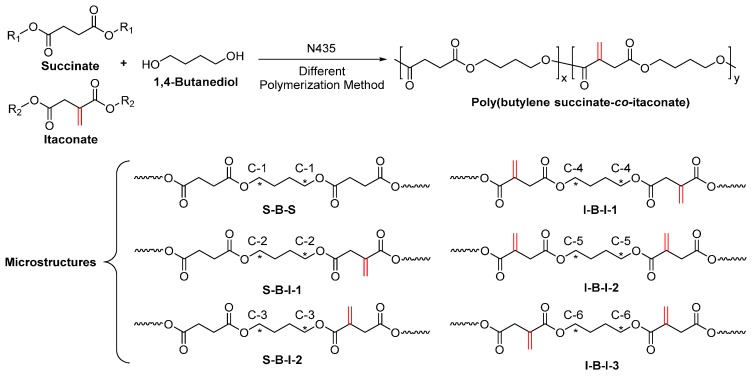
N435-catalyzed synthesis of poly(butylene succinate-*co*-itaconate) and the corresponding microstructures.

Recently, we investigated the N435-catalyzed polymerization of fully biobased poly(butylene succinate-*co*-itaconate) (PBSI) (Scheme 31) [264,265]. We found that the enzymatic polycondensation of succinic acid, itaconic acid, and 1,4-butanediol only yielded oligomers, with $\bar{M_{w}}$’s of around 500–1500 g/mol, despite different polymerization methods were used. By replacing the unactivated dicarboxylic acids with alkyl diesters, a series of PBSIs with various molar compositions and significant higher molecular weights were obtained, with $\bar{M_{w}}$’s of up to 28,300 g/mol. In addition, we found that: (1) the most suitable approach is azeotropic polymerization using the solvent mixture of cyclohexane and toluene, which results in PBSIs with high molecular weights and desirable chemical compositions; (2) high molecular weight PBSIs with <30 mol % of itaconate can be prepared by using the two-stage enzymatic polymerization in diphenyl ether; and (3) the two-stage enzymatic melt polymerization gives PBSIs with controllable chemical compositions but low molecular weights. Moreover, the ^13^C–NMR study revealed that different microstructures are present in PBSIs obtained from different polymerization methods. The formation of I-B-I-3 microstructures is crucial for synthesizing high molecular weight PBSIs with desired chemical compositions; and more I-B-I-3 microstructures can be produced by CALB in the solvent mixture of cyclohexane and toluene under an azeotropic condition.

**Scheme 32:**
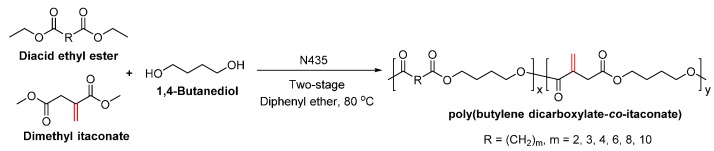
N435-catalyzed synthesis of poly(butylene dicarboxylate-*co*-itaconate) with diverse chemical structures [257].

However, by replacing diethyl succinate (C2) with the other diacid ethyl esters with relatively longer chain length (C3~C10), the two-stage enzymatic polymerization in diphenyl ether resulted in series of unsaturated aliphatic polyesters with desired molar compositions and high $\bar{M_{w}}$’s of up to 57,900 g/mol (Scheme 32) [257]. The molar percentage of itaconate in the unsaturated polyesters can be tailored from 0% to 35% by adjusting the feed ratio of itaconate; and all C=C bonds were well preserved in the resulting polyesters. We found that products with relatively lower molecular weights were generally obtained from the enzymatic polymerization at a higher feed ratio of itaconate; however, with diethyl dodecanedioate having the longest chain length (C10) among the tested diacid ethyl esters, higher molecular weight products were obtained at higher feed ratios of itaconate. Moreover, the obtained itaconate-based polyesters can be thermally cross-linked or photo-cured. By adjusting the diacid chain length and itaconate composition, the thermal and mechanical properties of the cured polyesters can be tuned.

### 8.3. Polyesters Derived from Long Chain Fatty Acids and their Derivatives

Long chain fatty acids may contain one or more C=C bonds within the backbones. The C=C bonds can be further modified to form other functional groups such as epoxy, thiol, and hydroxyl group [153,266], rendering fatty acid-based polyesters with diverse functionalities. Fatty acid-based polyesters can be used as thermoset resins, coating materials and biomaterials for biomedical applications, and so on [267,268,269].

**Scheme 33:**
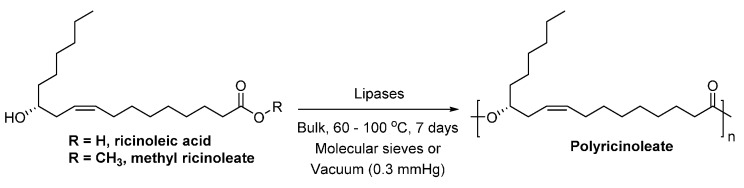
Lipase-catalyzed synthesis of polyricinoleate in bulk [270].

The pioneer work on the enzymatic polymerization with long chain fatty acids was reported by Matsumura et al. [270]. They investigated the lipase-catalyzed polymerization of ricinoleic acid/methyl ricinoleate in bulk (Scheme 33). Among the tested lipases, immobilized lipase PC showed the highest reactivity towards ricinoleic acid and methyl ricinoleate. The enzymatic polymerization with ricinoleic acid resulted in polyricinoleate with a $\bar{M_{w}}$ of up to 8500 g/mol. However, by replacing ricinoleic acid with methyl ricinoleate, polyricinoleate with a much higher $\bar{M_{w}}$ of up to 100,600 g/mol were produced.

**Scheme 34:**
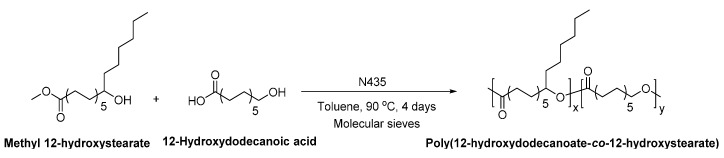
N435-catalyzed polycondensation of 12-hydroxydodecanoic acid and methyl 12-hydroxystearate [271].

Later, the N435-catalyzed synthesis of poly(12-hydroxydodecanoate-*co*-12-hydroxystearate) was studied by the same research group (Scheme 34) [271]. The $\bar{M_{w}}$, dispersity and reaction yield of the resulting *co*-polyesters were around 92,300–118,200 g/mol, 2.8–3.3, and 83%–88%, respectively. In addition, the molar percentage of 12-hydroxydodecanoate units in the final products can be tailored from 0% to 100% by adjusting the feed ratio.

**Scheme 35:**
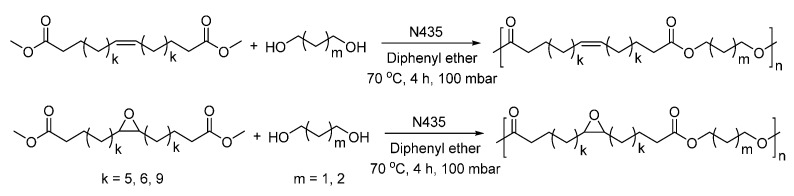
N435-catalyzed polycondensation of unsaturated or epoxidized ω-carboxy fatty acid methyl esters with alkane-α,ω-aliphatic diols [272].

**Scheme 36:**
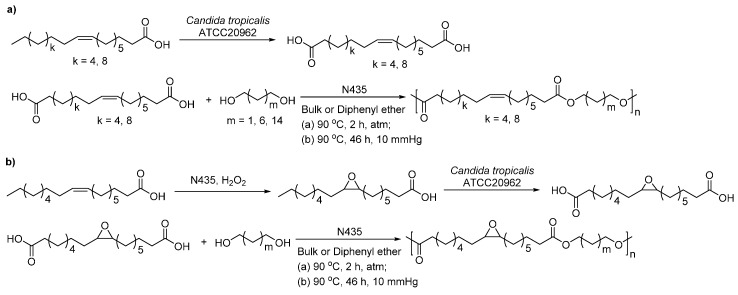
A two-step biocatalytic synthesis of biobased functional polyesters from ω-carboxy fatty acids and diols [273].

Biobased functional polyesters can be produced via enzymtic polymerization with unsaturated or epoxidized α,ω-carboxylic fatty acid derivatives [272,273]. The long chain unsaturated and epoxidized ω-carboxy fatty acid derivatives can be synthesized via chemical [272] or biocatalytic approaches [273]. The N435-catalyzed polycondensation of unsaturated or epoxidized ω-carboxy fatty acid methyl esters (C18, C20 and C26) with alkane-α,ω-aliphatic diols (C3 and C4) resulted in polyesters with $\bar{M_{w}}$’s, dispersities and reaction yields of up to 11,600 g/mol, 1.2–2.6, and 49%–84%, respectively (Scheme 35) [272]. In addition, the two-step biocatalytic approach gave biobased functional polyesters with $\bar{M_{w}}$’s and dispersities of around 25,000–76,000 g/mol, and around 2.0–3.1, respectively (Scheme 36) [273].

On the other hand, cutin and suberin are lipophilic macromolecules which are natural substances found in cell walls of higher plants as structural components. Cutin covers all the aerial surfaces of plants in the plant cuticle, while suberin is the main constituent of cork cells. Their fatty acid derivatives, such as long chain ω-hydroxyalkanoic acids, and α,ω-alkanedioic acids, and substituted ω-hydroxyalkanoic acids, are attractive biobased monomers for the synthesis of functional aliphatic polyesters [152,274].

**Scheme 37:**
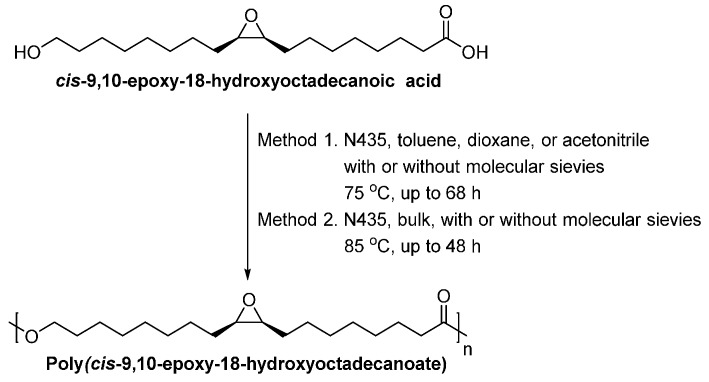
N435-catalyzed polymerization of *cis*-9,10-epoxy-18-hydroxyoctadecanoic acid in bulk or in an organic solvent.

Iversen et al. [275] did pioneer work on the enzymatic synthesis of suberin-based polyesters (Scheme 37). The N435-catalyzed polymerization with *cis*-9,10-epoxy-18-hydroxyoctadecanoic acid in toluene resulted in epoxy-functionalized polyesters with the highest molecular weights. The $\bar{M_{w}}$ and dispersity were 20,000 g/mol, and 2.2, respectively. In addition, even at a much shorter reaction time of 3 h, the solvent-free enzymatic polymerization in an open vial without any drying agents gave comparable high molecular weights products, with a $\bar{M_{w}}$ and a dispersity of 15,000 g/mol and 2.2, respectively.

**Scheme 38:**
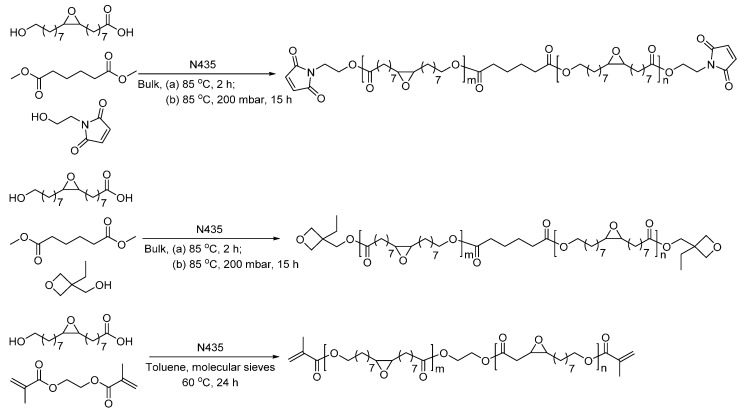
N435-catalyzed synthesis of bio-based oligoesters based on 9,10-epoxy-18-hydroxyoctadecanoic acid.

Recently, multifunctional, bio-based oligoester resins based on 9,10-epoxy-18-hydroxyoctadecanoic acid were enzymatically synthesized by using N435 as the catalysts (Scheme 38) [276,277]. The $\bar{M_{n}}$, dispersity, monomer conversion and reaction yield of the resulting oligoesters were around 900–1100 g/mol, 2.3–3.1, 95%–99%, and 82%–89%, respectively. Moreover, the functional end groups and the epoxy groups were well preserved after the enzymatic polymerization. The obtained oligoesters can undergo further modifications via different techniques such as Diels-Alder reactions, radical polymerization and ring-opening polymerization.

### 8.4. Glycerol-Based Polyesters

Glycerol-based aliphatic polyesters can be used as thermosets like shape memory materials; and have found potential applications in biomedical and pharmaceutical fields, for example, they can be used as carriers for drug delivery, sealants or coatings for tissue repair, and agents for antibacterial applications [278].

**Scheme 39:**
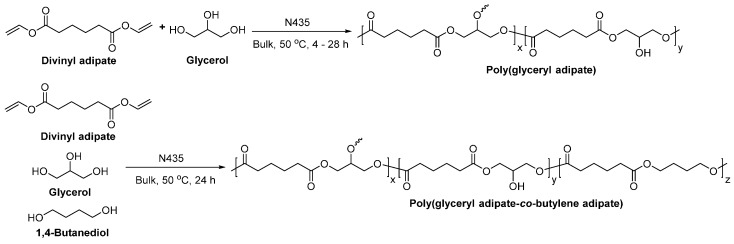
N435-catalyzed polymerization of divinyl adipate and glycerol with or without 1,4-butanediol in bulk [279].

The N435-catalyzed polymerization of divinyl adipate and glycerol in bulk yielded poly(glyceryl adipate) with a $\bar{M_{w}}$ and dispersity of up to 10,400 g/mol and 2.3–3.1, respectively (Scheme 39) [279]. MALDI-ToF MS analysis suggested that linear polyesters with hydroxyl substituents were mainly produced and no polymer network was formed. The number of hydroxyl groups per repeating units was around 0.8–0.9; and the pendant groups of the synthetic poly(glyceryl adipate) consisted of 90%–95% of secondary and 5%–10% of primary hydroxyl groups.

**Scheme 40:**
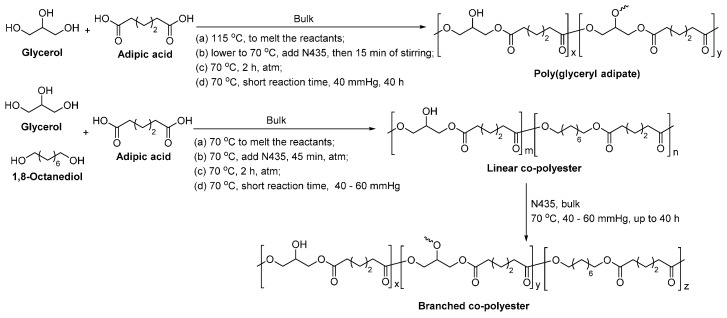
N435-catalyzed polymerization of glycerol and adipic acid with or without 1,8-octanediol in bulk [280,281,282].

By replacing the activated divinyl adipate with unactivated adipic acid, the N435-catalyzed enzymatic polycondensation also resulted in poly(glyceryl adipate), with a slight low $\bar{M_{w}}$ of 3700 g/mol and a dispersity of 1.4 (Scheme 40) [280].

**Scheme 41:**
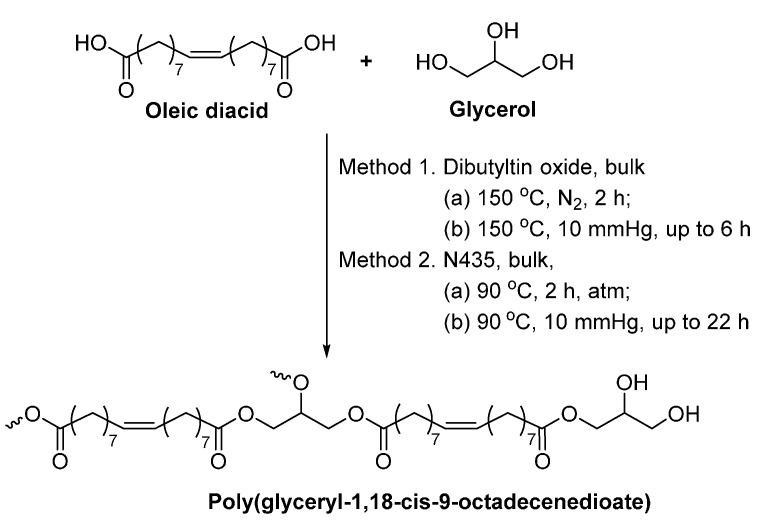
Chemical and enzymatic synthesis of poly(glyceryl-1,18-*cis*-9-octadecenedioate) [283].

Poly(glyceryl-1,18-*cis*-9-octadecenedioate) was successfully produced via the N435-catalyzed polycondensation of 1,18-*cis*-9-octadecenedioic (oleic diacid) and glycerol in bulk (Scheme 41) [283]. At a molar monomer feed ratio of 1.0:1.0, the $\bar{M_{n}}$ and dispersity of the obtained polyesters were up to 9100 g/mol, and around 3.3–3.4, respectively. However, the percentage of dendritic glycerol units (Den %) was quite low, around 13%–16%. By increasing the molar feed ratio of oleic diacid and glycerol from 1.0:1.0 to 1.0:1.5, the resulting polyesters possessed a similar $\bar{M_{n}}$ and dispersity, but a significant higher Den % (~31%). In contrast, gelation was observed in the polymerization catalyzed by dibutyltin oxide.

Moreover, several glycerol-based *co*-polyesters were successfully produced via lipase-catalyzed polycondensation.

The enzymatic *co*-polymerization of divinyl adipate, glycerol and 1,4-BDO gave poly(glyceryl adipate-*co*-butylene adipate) (Scheme 39) [279]. The hydroxyl number of the obtained *co*-polyesters can be well controlled by adjusting the amount of 1,4-BDO in the reaction mixture.

Poly(octamethylene adipate-*co*-glyceryl adipate) were successfully produced via the N435-catalyzed *co*-polymerization of glycerol, adipic acid and 1,8-octanediol in bulk (Scheme 40) [280,281,282], with $\bar{M_{n}}$’s of up to 75,600 g/mol. The ^13^C–NMR study indicated that the obtained polyesters were highly branched but had few interchain cross-links; and the degree of branching and molecular weights can be controlled by altering the reaction time and molar feed ratio of monomers [282]. In addition, due to the regio-selectivity of N435, the enzymatic polymerization gave linear polyesters at short reaction times (≤18 h) but yielded highly branched polyesters at a long reaction time (42 h). Moreover, with respect to esterifications, N435 showed 77% to 82% of the regio-selectivity towards the primary hydroxyl groups of glycerol and this was independent of the amount of glycerol in the reaction media.

**Scheme 42:**
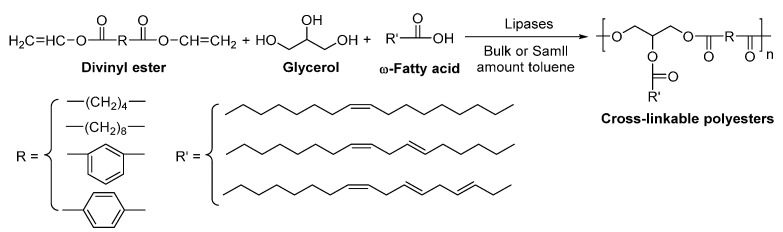
N435-catalyzed *co*-polymerization of divinyl esters, glycerol and ω-fatty acids [284,285].

The enzymatic *co*-polymerization of divinyl esters, glycerol and ω-fatty acids were also investigated, using lipases as biocatalysts (Scheme 42) [284,285]. Among the tested lipases, N435 showed the highest catalytic activity. The enzymatic polymerization yielded biodegradable cross-linkable polyesters with $\bar{M_{n}}$’s of up to 8500 g/mol. The obtained polyesters were thermally cross-linked, which resulted in transparent polymeric films with high-gloss surfaces.

**Scheme 43:**
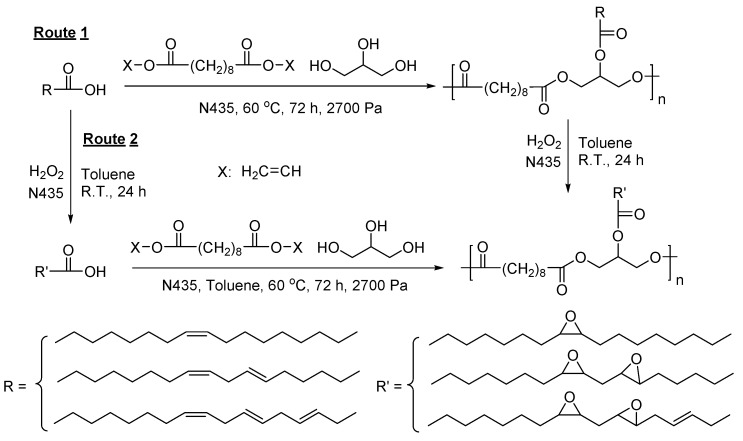
N435-catalyzed synthesis of epoxide-containing polyesters from polycondensation of divinyl sebacate, glycerol and vegetable oil-based fatty acids [286].

Later, the same research group reported the synthesis of epoxide-containing, glycerol-based *co*-polyesters via N435-catalzyed polymerization via two routes (Scheme 43) [286]. The first route yielded corresponding polyesters with $\bar{M_{n}}$’s, dispersities and epoxidation ratios of around 3300–7900 g/mol, 1.3–1.6, and 76%–96%, respectively; and the second route gave relatively higher values of $\bar{M_{n}}$, dispersity and epoxidation ratio, which were around 4200–6500 g/mol, 1.9–2.1, and 88%–94%, respectively.

**Scheme 44:**
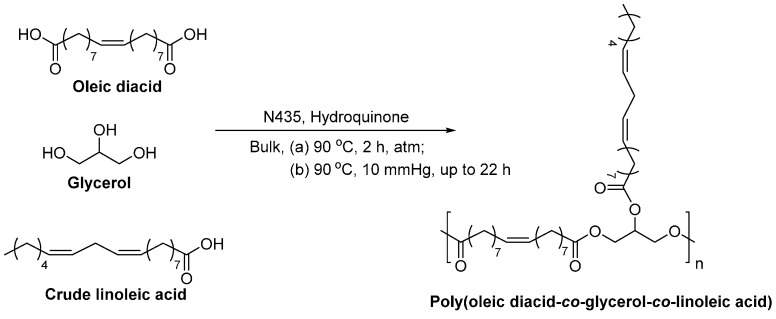
N435-catalyzed synthesis of poly(oleic diacid-*co*-glycerol-*co*-linoleic acid) in bulk [287].

Recently, polymeric triglyceride analogs, poly(oleic diacid-*co*-glycerol-*co*-linoleic acid)s, were prepared via the N435-catalyzed polycondensation (Scheme 44) [287]. By varying the molar feed ratio of oleic diacid, glycerol and crude linoleic acid from 1.0:1.0:1.0 to 1.0:1.0:1.33, the $\bar{M_{n}}$ and dispersity of the obtained products decreased from 12,300 to 6300 g/mol, and 6.3 to 1.7, respectively, whereas the degree of tri-substituted units increased from 18% to 100%. In addition, when the molar feed ratio of oleic diacid, glycerol and crude linoleic acid was 1.0:1.0:0.67, all monomers were converted to polymers after 8 h reaction; and the $\bar{M_{n}}$ and degree of tri-substituted units of the resulting *co*-polyesters reached 9500 g/mol, and 64%, respectively.

### 8.5. Sweet Polyesters Derived from Carbohydrates

#### 8.5.1. Sugar and Sugar Alcohol-Based Polyesters

Sugars and sugar alcohols can be used as starting materials for the production of biobased linear and branched functional polyesters which have various potential applications, for example, they can be used as coating materials, biodegradable and bioresorbable polymers, and optically active polymers [112].

It is difficult to synthesize polyesters with sugar and sugar alcohol units via conventional techniques, as tedious protection and de-protection steps are normally required, to prevent the gelation during the polymerization. However, these multiple-functional monomers can be directly polymerized via enzymatic polymerization, due to the highly regio-selectivity of the enzymes.

**Scheme 45:**
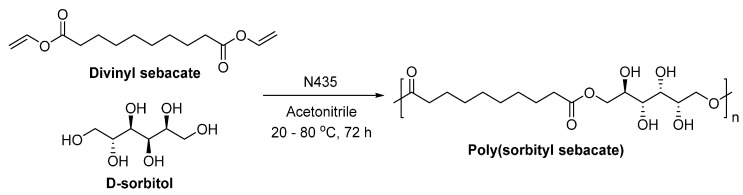
N435-catalyzed polymerization of d-sorbitol with divinyl sebacate [288].

The lipase-catalyzed polymerization with d-sorbitol was first reported by Kobayashi et al. (Scheme 45) [288]. Sorbitol-based polyesters with $\bar{M_{n}}$’s of around 3000–12,000 g/mol were produced in moderate yields (around 40%–85%). Moreover, ^1^H– and ^13^C–NMR study indicated that the primary hydroxyl groups of d-sorbitol were exclusively esterified during the enzymatic polymerization. However, to compensate the low catalytic reactivity of N435 in the polar aprotic solvent acetonitrile, activated divinyl sebacate and high concentration of N435 (76 wt %) were used.

In addition, divinyl sebacate and other activated monomers including bis(2,2,2-trifluoroethyl)malonate, bis(2,2,2-trifluoroethyl)glutarate and divinyl adipate, were enzymatically polymerized with disaccharides (sucrose, trehalose, and lactose), d-sorbitol and d-mannitol in acetonitrile. [289] It was found that N435 was able to differentiate the five tested carbohydrates, as the molecular weights of the resulting polyesters showed the following array: disaccharide-based polyesters < mannitol-based polyesters < sorbitol-based polyesters.

**Scheme 46:**
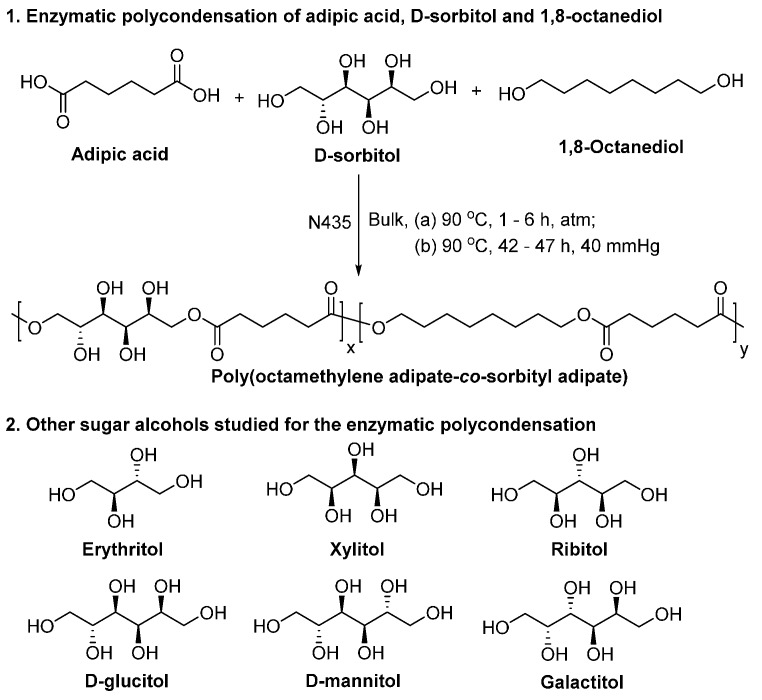
N435-catalyzed *co*-polymerization of adipic acid and 1,8-octanediol with different sugar diols in bulk [280,281,290].

Unactivated diacid monomers were also used as starting materials for the enzymatic synthesis of sorbitol-based polyesters. Gross et al. [280,281] reported the N435-catalyzed polycondensation of d-sorbitol, 1,8-octanediol and adipic acid in bulk (Scheme 46). Poly(sorbityl adipate) and poly(octamethylene adipate-*co*-sorbityl adipate)s were produced, with $\bar{M_{n}}$’s and dispersities of around 7000–20,300 g/mol, and 1.6–3.3, respectively. In addition, the molar percentage of sorbityl units in the *co*-polyesters can be tunable from 0% to 100%. Moreover, the NMR analysis revealed that N435 showed highly regio-selectivity (≥85% ± 5%) towards the primary hydroxyl groups of d-sorbitol at 1- and 6-positions [280].

In the same laboratory, the enzymatic polycondensation of adipic acid and 1,8-octanediol with different sugar alcohols was investigated (Scheme 46) [290]. Sweet *co*-polyesters with $\bar{M_{w}}$’s ranging from 11,000 and 73,000 g/mol were obtained; and no correlation was observed between the reactivity of the tested sugar diols and their chain length. However, N435 showed the highest reactivity towards d-mannitol; and the *co*-polyester containing d-mannitol units possessed the highest degree of branching.

**Scheme 47:**
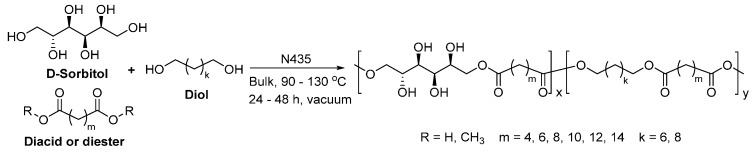
N435-catalyzed *co*-polymerization of d-sorbitol, alkane-α,ω-aliphatic linear diols and diacids or diesters in bulk.

Recently, sorbitol-based, hydroxy-functional polyesters were successfully synthesized via the N435-catalyzed *co*-polymerization (Scheme 47) [291]. The $\bar{M_{n}}$ of the resulting *co*-polyesters was successfully controlled at around 4000–8000 g/mol, by tuning the reaction time, enzyme concentration and reaction stoichiometry. However, only maximum 53% of the added d-sorbitol was incorporated into the final products, even though its feed ratio was quite low (≤5 mol %). Besides, the obtained polyesters displayed suitable properties for being used as solvent-borne coating resins.

#### 8.5.2. Polyesters Based on Rigid Sugar Derivatives

DAHs (isosorbide, isomannide and isoidide) and the diacetalized monomers 2,3:4,5-di-*O*-methylene-galactaric acid (Glux diacid) and 2,4:3,5-di-*O*-methylene-d-glucitol (Glux diol), are rigid compounds which are derived from sugars. They are good candidates for polyester synthesis, rendering polyesters with high values of *T*_g_ and better thermal stability.

**Scheme 48:**
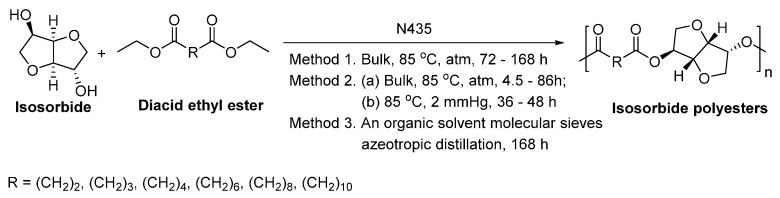
N435-catalyzed synthesis of isosorbide polyesters [292].

Isosorbide was enzymatically polymerized with various aliphatic diacid ethyl esters in the presence of N435 (Scheme 48), as reported by Catalani et al. [292]. The solvent-free enzymatic polymerization gave low molecular weight poly(isosorbide adipate) ($\bar{M_{w}}$ ≤ 3800 g/mol), as the hydroxyl groups of isosorbide can be condensed by N435. However, high molecular weight isosorbide polyesters with $\bar{M_{w}}$’s of up to 40,000 g/mol were produced via the enzymatic polymerization by azeotropic distillation; and significantly higher molecular weight products can be obtained by decreasing the concentration of reactants in the reaction media. Meanwhile, it was found that the suitable solvents for the enzymatic polymerization were cyclohexane, cyclohexane/benzene (6:1, *v/v*) and cyclohexane/toluene (6:1, *v/v*). Furthermore, the enzymatic azeotropic polymerization in cyclohexane/toluene gave isosorbide polyesters with a higher $\bar{M_{w}}$ when the chain length of the tested aliphatic diacid ethyl esters increased from C4 to C6. However, by further increasing the chain length from C6 to C8, C10 and 12, isosorbide polyesters with lower molecular weights were produced.

**Scheme 49:**
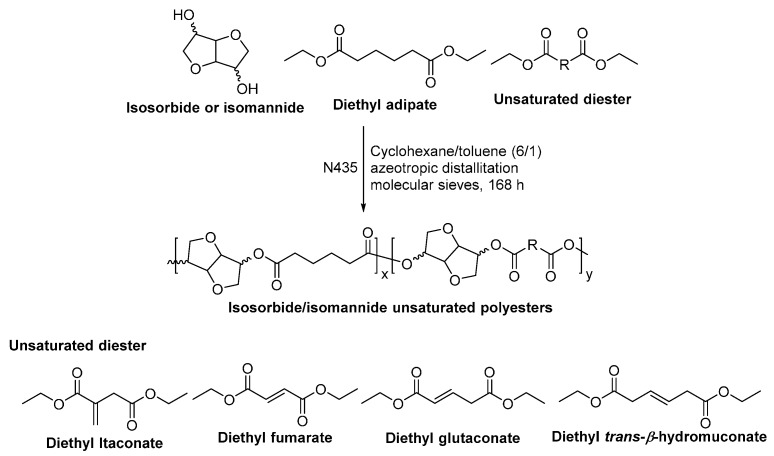
N435-catalyzed azeotropic polycondensation of isosorbide/isomannide, diethyl adipate and fractions of different unsaturated diesters.

The same research group also investigated the N435-catalyzed azeotropic polycondensation of isosorbide/isomannide and diethyl adipate with different fractions of unsaturated diesters (Scheme 49) [293]. No homo-polyesters and *co*-polyesters containing itaconate units were obtained via the enzymatic polymerization. However, high molecular weight isosorbide/isomanide-based unsaturated *co*-polyesters were produced from the enzymatic azeotropic polymerization with the other tested unsaturated diesters, with $\bar{M_{w}}$’s of up to 15,900 g/mol. Moreover, Michael additions of water to C=C bonds occurred during the enzymatic polymerization; and the corresponding polymers can undergo additional reactions through hydroxyl pendant groups. Besides, the enzymatic polymerization involving isosorbide gave much higher molecular weights products, which suggested that N435 prefers isosorbide over isomannide. This is however in contract with the result reported by Boeriu et al. [294]. They investigated the N435-catalyzed polymerization of succinic acid with isomannide, isosorbide or isoidide in toluene/*tert*-butanol; and found that N435 showed preference for isomannide over isosorbide and over isoidide. They attributed this to the preference of N435 for the *endo*-hydroxyl groups, which is due to the fact that the transition state of esters with *ex*o-hydroxyl groups does not form all the required hydrogen bonds for catalysis.

**Scheme 50:**
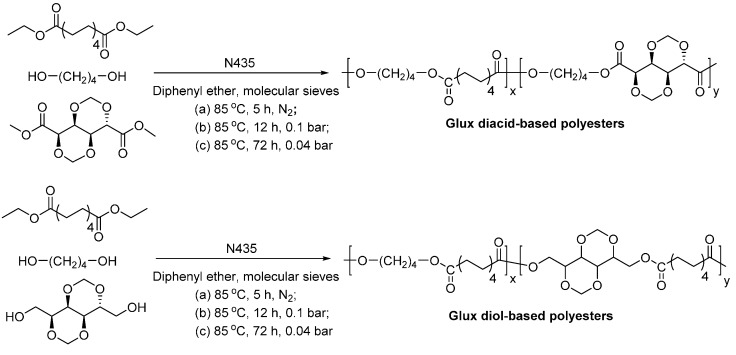
N435-catalyzed synthesis of polyesters containing glucitylene or glucarate units [295].

A series of Glux diacid- and Glux diol-based polyesters were synthesized via the N435-catalyzed polycondensation of diethyl sebacate and 1,4-BDO with Glux diacid/Glux diol (Scheme 50) [295]. The $\bar{M_{w}}$, reaction yield and intrinsic viscosity of the resulting polyesters were around 10,000 g/mol, 30%–70%, and 0.3–0.44 dL/g, respectively. In addition, the molecular weights and reaction yields of the obtained Glux diacid-based polyesters were lower than those of the synthetic Glux diol-based polyesters; and these two parameters decreased monotonically with increasing the Glux content in both Glux diacid- and Glux diol-based polyesters. Moreover, no polyester was obtained from the enzymatic polymerization of Glux diacid and 1,4-BDO. This can be explained by the bulky bicyclic structure next to the carboxylate groups, which hinders the access of this group to the active site of the enzyme CALB. Furthermore, all the synthetic polyesters possess random microstructures.

### 8.6. Biobased Polyamides

At present, studies related to the enzymatic synthesis of biobased synthetic polyamides are scarcer. A few potentially biobased aliphatic polyamides, such as nylon 4,10, nylon 6,10, and nylon 8,10, can be synthesized via lipase-catalyzed polymerization. However, the molecular weights of the obtained polyamides were quite low.

**Scheme 51:**
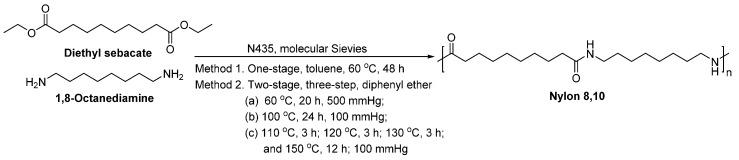
N435-catalyzed synthesis of nylon 8,10 from polycondensation of diethyl sebacate and 1,8-octanediamine [197].

Landfester et al. [197] studied the N435-catalyzed polycondensation of diethyl sebacate and 1,8-octanediamine (Scheme 51). The enzymatic polymerization gave nylon 8,10 with $\bar{M_{n}}$’s of around 2000–5000 g/mol.

**Scheme 52:**
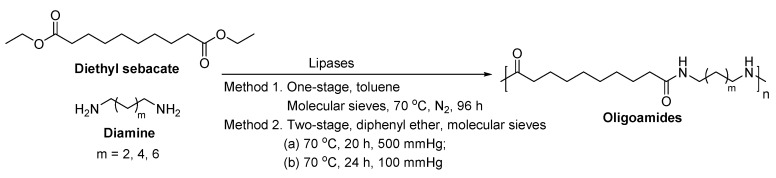
Lipase-catalyzed polycondensation of diethyl sebacate and diamines.

In our group, oligomers including nylon 4,10, nylon 6,10, and nylon 8,10 were produced via the lipase-catalyzed polymerization of diethyl sebacate with different diamines, with a $\bar{DP_{\text{max}}}$ of up to 16 (Scheme 52) [235].

### 8.7. Biobased Furan Polyesters and Furan Polyamides

Furan polymers are not new polymers. In the late 1970s, poly(hexamethylene furanoate), a furan polyester, was synthesized by Moore and Kelly [296,297]; and various furan polyesters were successfully prepared by Ballauff et al. [298], Gandini et al. [299], and Okada et al. [300] since 1990s.

In recent years, the research on FDCA-based polyesters and polyamides is booming, due to the fast development of biobased FDCA and the broad potential applications of FDCA-based polymers [16]. The FDCA-based polymers are promising sustainable aromatic polymer alternatives, and FDCA-based polymers possess similar or even better properties than their petrol-base counterparts. For example, recent studies suggested that poly(ethylene furanoate) (PEF) possesses better barrier properties compared to PET: PEF shows surprisingly large reductions in CO_2_ permeability (19×), O_2_ permeability (11×) and diffusivity (31×) [301,302].

At present, FDCA-based polyesters and polyamides are predominately synthesized via melt polycondensation at elevated temperatures of around 200 °C. However, decarboxylation of FDCA takes place at around 195 °C and other side-reactions may occur at such elevated temperatures [16,303,304,305], which may lead to the discoloration of the resulting polymers and the formation of low molecular weight products. However, these drawbacks could be circumvented by using enzyme catalysts.

**Scheme 53:**
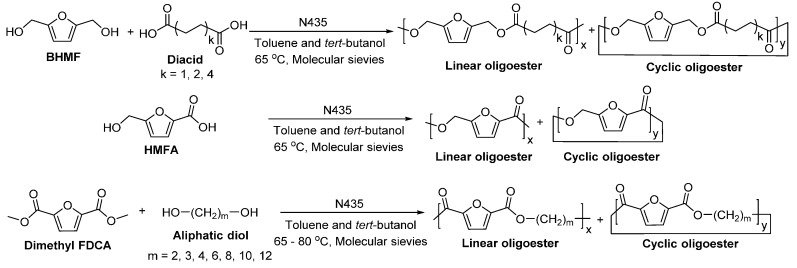
N435-catalyzed polymerizations with furan monomers: bis(hydroxymethyl)furan (BHMF), 5-hydroxymethyl-2-furancarboxylic acid (HMFA) and dimethyl 2,5-Furandicarboxylic acid (FDCA) [306,307].

The N435-catalyzed polymerization with dimethyl 2,5-furandicarboxylate (dimethyl FDCA)/BHMF/5-hydroxymethyl-2-furancarboxylic acid (HMFA) was reported by Habeych N. [306] and Boeriu et al. [307], using a one-stage method in the mixture of toluene and *tert*-butanol. However, only a mixture of linear and cyclic furan oligomers were produced (Scheme 53).

**Scheme 54:**
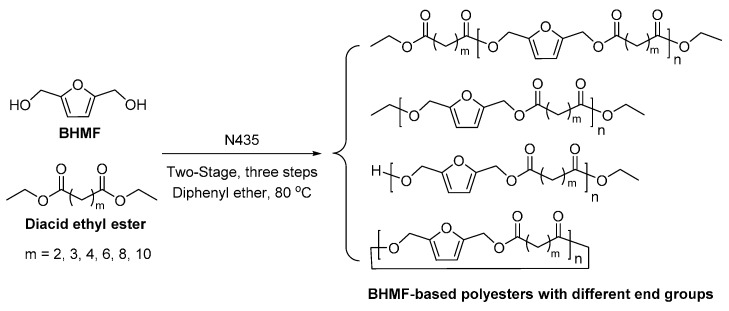
N435-catalyzed polycondensation of BHMF and diacid ethyl esters via a two-stage, three step method in diphenyl ether at 80 °C [308].

Recently, we studied the N435-catalyzed polymerization of BHMF and various diacid ethyl esters, using the two-stage, three step method (Scheme 54) [308]. BHMF-based polyesters with low molecular weights were produced, with $\bar{M_{w}}$’s of around 1800–2900 g/mol. The polymerization kinetic study and MALDI-ToF MS analysis revealed that ether end groups were formed during the enzymatic polymerization, which led to the low molecular weights.

**Scheme 55:**
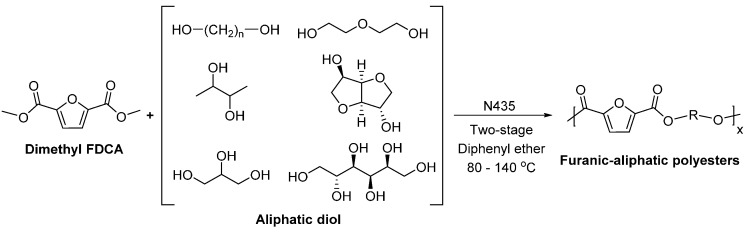
N435-catalyzed polycondensation of dimethyl FDCA and aliphatic diol via a two-stage method in diphenyl ether [309].

FDCA-based furanic-aliphatic polyesters were successfully produced via the enzymatic polymerization of dimethyl FDCA with various aliphatic diols, using a two-stage method in diphenyl ether at 80–140 °C (Scheme 55) [309]. The obtained polyesters reached a very high $\bar{M_{w}}$ of up to 100,000 g/mol, which is normally hard to achieve by enzymatic polymerization. For the first time we demonstrated that enzymatic polymerizations are capable of producing high molecular weight FDCA-based polyesters, which have been primarily synthesized via step-growth polymerization using organometallic catalysts at elevated temperatures around 150–280 °C. Moreover, we found that CALB prefers alkane-α,ω-aliphatic linear diols of > 3 carbons. Furthermore, the FDCA-based furanic-aliphatic polyesters possess similar crystalline and thermal properties compared to their petrol-based counterparts, semi-aromatic polyesters.

**Scheme 56:**
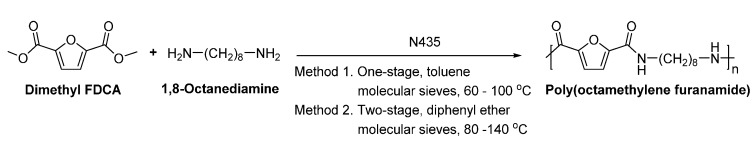
N435-catalyzed polycondensation of dimethyl FDCA and 1,8-octanediamine via a one-stage method in toluene and a temperature varied two-stage method in diphenyl ether [310].

Furthermore, high molecular weight FDCA-based furanic-aliphatic polyamides were produced from the enzymatic polycondensation of dimethyl FDCA and 1,8-ODA, using a one-stage method or a temperature-varied two-stage method (Scheme 56) [310]. The FDCA-based furanic-aliphatic polyamides can be used as a promising sustainable alternatives to petrol-based polyphthalamides (semi-aromatic polyamides) and be applied as thermoplastic engineering polymers and high performance materials. The enzymatic polymerization resulted in poly(octamethylene furanamide) (PA 8,F) with a very high $\bar{M_{w}}$ of up to 54,000 g/mol. This is the first time that FDCA-based polyamides are successfully produced via enzymatic polymerization; and the molecular weights of the obtained PA 8,F are much higher than those produced via melt-polycondensation, the primarily synthesis approach for semi-aromatic polyamides, at elevated temperatures usually above 200 °C. Moreover, the obtained PA 8,F possesses a similar *T*_g_ and similar crystal structures, a comparable *T*_d_, but a lower *T*_m_, compared to its petrol-based counterpart, poly(octamethylene terephthalamide) (PA 8,T).

## 9. Conclusions and Outlook

Enzymatic polymerization is proven to be a powerful and versatile approach for the production of biobased polyesters and polyamides with different chemical compositions (aliphatic and semi-aromatic polymers), varied architectures (linear, branched and hyperbranched polymers), and diverse functionalities (pendant hydroxyl groups, carbon–carbon double bonds, epoxy groups, and so on). Among the enzymes studied for biobased polyester and polyamide synthesis, CALB, especially its immobilized form N435, shows broad monomer adaptability, stable and excellent catalytic performance, and great tolerance of various conditions. Moreover, with the mild synthetic conditions, non-toxic and renewable enzyme catalysts, and sustainable starting materials, synthesis of biobased polymers via enzymatic polymerizations provides an opportunity for achieving green polymers and a future sustainable polymer industry, which will eventually play an essential role for realizing and maintaining a green and sustainable society.

However, this approach also possesses some limitations and disadvantages:

(1) the atom efficiency is low when ester derivatives rather than acids are used;
(2) non-ecofriendly solvents including diphenyl ether and toluene are commonly used;
(3) long polymerization times are required for achieving high molecular weights;
(4) high reaction temperatures at around 100–140 °C were applied for enzymatic synthesis of polymers having a high *T*_m_ and low solubility; and the catalytic reactivity of enzymes decreases significantly at such elevated temperatures;
(5) the price of enzyme catalysts is still quite high;
(6) enzymatic polymerizations involving monomers with short chain length like 1,3-propanediol, monomers with secondary hydroxyl groups such as isosorbide and 2,3-butanediol, and polyols, generally result in low molecular weight products;
(7) the purify and price of biobased monomers remain a concern;
(8) last but certainly not least, only limited variety of biobased monomers are currently commercially available.

Therefore, more efforts are required to address these problems. For example, acids can be used to improve the atom efficiency, green solvents such as ionic liquids and supercritical CO_2_ can be employed as the reaction media, more robust and thermal stable enzymes should be developed for enzymatic polymerizations, improved and optimal processes should be explored for the production of diverse biobased monomers with high purity and low price, and so on.

Although numerous polyesters and polyamides are readily produced by using free lipases and immobilized lipases as the catalysts, the explanations for the different polymerization results are not clear yet. This could be an interest topic for the future research.

Noteworthy is that many experimental results reveal that lipase-catalyzed polymerizations involving structurally similar monomers afford polymers with different compositions and varied molecular weights. This could be attributed to the synergistic effect caused by many reasons, for example, the specificity and selectivity of lipases towards different monomers, the physical properties of the starting materials (purity, melting temperature, and miscibility and solubility in the reaction media), the physical properties of the resulting intermediates and the final products (glass transition temperature, melting temperature, crystallization ability, and miscibility and solubility in the reaction media), the enzymatic polymerization conditions, and so on. However, such synergistic effect has not been fully understood yet, which requires systematic studies in the future. Besides, it would be of great interest to employ computer simulations to study the specificity and selectivity of lipases for the monomers in the enzymatic polymerization, as well as, the enzymatic polymerization mechanism.

At present, enzymatic polymerizations have already been poised for use in commercial process to prepare polymers targeted for cosmetic and medical applications. However, polymers including biobased polymers are still predominately produced via conventional approaches. Due to the fast development of biotechnologies and enzymatic polymerization techniques, and the increased realization of the great benefits that enzymatic polymerizations and biobased monomers have to offer, there will be more highly value-added specialty biobased polymers produced commercially via biocatalytic approach in the near future. However, for the production of biobased commodity polymers, engineering plastics and high performance polymers, the commercial enzymatic process is promising but still has a long way to go, considering the high efficiency and low cost of the current pathways to the petrol-based counterparts.

## Figures and Tables

**Table 1:** A selected list of commercially available biobased polyesters and their manufacturers.

Biobased Polyester	Biosourcing (%) ^a^	Manufacturer	Trademark
PLA	up to 100	NatureWorks (Minnetonka, MN, USA)	Ingeo™, NatureWorks^®^
		Synbra (Etten-Leur, The Netherlands)	BioFoam^®^
		Zhejiang Hisun Biomaterials Biological Engineering (Taizhou, Zhejiang, China)	REVODE 100 and 200 series
		Nantong Jiuding Biological Engineering (Rugao, Jiangsu, China)	-
		Teijin (Chiyoda, Tokyo, Japan, Japan)	BIOFRONT™
		Mitsui Chemicals (Minato, Tokyo, Japan)	LACEA^®^
		Futerro (Celles, Belgium)	Futerro^®^
		Corbion Purac (Amsterdam, The Netherlands)	LX175, L175, L130, L105, D070
PHAs	100	Metabolix (Cambridge, MA, USA) and ADM (Decatur, IL, USA)	Mirel™
		MHG (Bainbridge, GA, USA)	Nodax™
		Bio-on (San Giorgio di Piano, Bologna, Italy)	MINERV-PHA™
		Tianjin Green Biosciences (Tianjin, China)	GreenBio
		Kaneka (Tokyo, Japan)	Kaneka PHBH
		Tianan Biological Materials (Ningbo, Zhejiang, China)	ENMAT™
		PHB Industrial S/A (Serrana, Brazil)	BIOCYCLE^®^
PBS	50	PTT MCC Biochem ( Chatuchak, Bangkok, Thailand)	BioPBS™
		Showa Denko K.K. (Tokyo, Japan)	Bionolle™
		Mitsubishi Chemical (Chiyoda-ku, Tokyo, Japan)	GS Pla^®^
PEF	100	Avantium (Geleen, The Netherlands)	-
PET	up to 30	Coca Cola (Atlanta, GA, USA)	PlantBottle™
		Toyota Tsusho Corporation (Nagoya, Aichi Prefecture, Japan)	GLOBIO^®^
PTT	37	DuPont (Wilmington, DE, USA)	Sorona^®^
	up to 35		Biomax^®^
PBAT	30–70	Novamont (Novara, Italy)	Origo-Bi™
		BASF (Ludwigshafen, Germany)	Ecoflex^®^ FS
*Co*-polyester	9–30	SK Chemicals (Seongnam-si, Gyeonggi-do, Korea)	ECOZEN^®^
*Co*-polyester	-	DuPont (Wilmington, DE, USA)	Biomax^®^

^a^ Biosourcing (%): the percentage of carbon originating from biomass sources among the total organic carbon.

**Table 2:** A selected list of commercially available biobased polyamides and their manufacturers.

Biobased Polyamide	Biosourcing (%) ^a^	Manufacturer	Trademark
Nylon 4,10 (PA 4,10)	100	DSM (Heerlen, The Netherlands)	EcoPaXX^®^
Nylon 6,10 (PA 6,10)	63	BASF (Ludwigshafen, Germany)	Ultramid^®^ S Balance
		EMS-GRIVORY (Domat/Ems, Switzerland)	Grilamid^®^ 2S
		Evonik (Essen, Germany)	VESTAMID^®^ Terra HS
		Solvay (Rhodia) (Brussels, Belgium)	Technyl^®^ eXten
		DuPont (Wilmington, DE, USA)	Zytel^®^ RS LC3030
		Arkema (Colombes, France)	Rilsan^®^ S
		Suzhou Hipro Polymers (Suzhou, Jiangsu, China)	Hiprolon^®^ 70
Nylon 10,10 (PA 10,10)	100	EMS-GRIVORY (Domat/Ems, Switzerland)	Grilamid^®^ 1S
		Evonik (Essen, Germany)	VESTAMID^®^ Terra DS
		DuPont (Wilmington, DE, USA)	Zytel^®^ RS LC1000
		Arkema (Colombes, France)	Rilsan^®^ T
		Suzhou Hipro Polymers (Suzhou, Jiangsu, China)	Hiprolon^®^ 200, Hiprolon® 211
Nylon 10,12 (PA 10,12)	45	Evonik (Essen, Germany)	VESTAMID^®^ Terra DD
		Suzhou Hipro Polymers (Suzhou, Jiangsu, China)	Hiprolon^®^ 400
Nylon 11 (PA 11)	100	Arkema (Colombes, France)	Rilsan^®^ PA11
		Suzhou Hipro Polymers (Suzhou, Jiangsu, China)	Hiprolon^®^ 11
PA 10,T	50	EMS-GRIVORY (Domat/Ems, Switzerland)	Grilamid^®^ HT3
		Evonik (Essen, Germany)	VESTAMID^®^ HTplus M3000
Polyphthalamide (PPA)	>70	Arkema (Colombes, France)	Rilsan^®^ HT
Transparent polyamide	54	Arkema (Colombes, France)	Rilsan^®^ Clear G830 Rnew
*Co*-polyamide	Tailored, up to 100	Arkema (Colombes, France)	Platamid^®^ Rnew
Polyamide	High Bio-Content	EMS-GRIVORY (Domat/Ems, Switzerland)	Grilamid^®^ TR

^a^ Biosourcing (%): the percentage of carbon originating from biomass sources among the total organic carbon.

**Table 3:** Enzymes and typical examples for their use in polymer synthesis, and typical polymers synthesized via enzymatic polymerization [200].

Enzyme class	Reaction catalyzed	Typical enzymes	Typical polymers
EC 1. Oxidoreductases	Oxidation/Reduction AH_2_ + B $\overset{\;}{\to }$ A + BH_2_	Peroxidase Laccase	Polyanilines Polyphenol Polystyrenes Poly(methyl methacrylate)
EC 2. Transferases	Group transfer A-X + B $\overset{\;}{\to }$ A + B-X	PHA synthase Hyaluronan synthase Phosphorylase	Polyesters Hyaluronan Amylose
EC 3. Hydrolases	Hydrolysis by H_2_O A-B + H_2_O $\overset{\;}{\to }$ AH + BOH	Lipase Cellulase Hyaluronidase Papain	Polyesters Polyamides Cellulose (Oligo)peptides Glycosaminoglycan
EC 6. Ligases	Bond formation requiring triphosphate 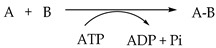	Cyanophycin synthetase	Cyanophycin
